# Mapping the strain-stiffening behavior of the lung and lung cancer at microscale resolution using the crystal ribcage

**DOI:** 10.3389/fnetp.2024.1396593

**Published:** 2024-07-10

**Authors:** Robert LeBourdais, Gabrielle N. Grifno, Rohin Banerji, Kathryn Regan, Bela Suki, Hadi T. Nia

**Affiliations:** Department of Biomedical Engineering, Boston University, Boston, MA, United States

**Keywords:** crystal ribcage, elastography, multiscale modeling, *ex vivo*, finite-element analysis, network physiology, cancer, strain-stiffening 27

## Abstract

Lung diseases such as cancer substantially alter the mechanical properties of the organ with direct impact on the development, progression, diagnosis, and treatment response of diseases. Despite significant interest in the lung’s material properties, measuring the stiffness of intact lungs at sub-alveolar resolution has not been possible. Recently, we developed the crystal ribcage to image functioning lungs at optical resolution while controlling physiological parameters such as air pressure. Here, we introduce a data-driven, multiscale network model that takes images of the lung at different distending pressures, acquired via the crystal ribcage, and produces corresponding absolute stiffness maps. Following validation, we report absolute stiffness maps of the functioning lung at microscale resolution in health and disease. For representative images of a healthy lung and a lung with primary cancer, we find that while the lung exhibits significant stiffness heterogeneity at the microscale, primary tumors introduce even greater heterogeneity into the lung’s microenvironment. Additionally, we observe that while the healthy alveoli exhibit strain-stiffening of ∼1.75 times, the tumor’s stiffness increases by a factor of six across the range of measured transpulmonary pressures. While the tumor stiffness is 1.4 times the lung stiffness at a transpulmonary pressure of three cmH_2_O, the tumor’s mean stiffness is nearly five times greater than that of the surrounding tissue at a transpulmonary pressure of 18 cmH_2_O. Finally, we report that the variance in both strain and stiffness increases with transpulmonary pressure in both the healthy and cancerous lungs. Our new method allows quantitative assessment of disease-induced stiffness changes in the alveoli with implications for mechanotransduction.

## 1 Introduction

Altered stiffness is one of the four physical hallmarks of cancer (Nia et al., 2020a; Zhang et al., 2023a), with implications for the development, progression, diagnosis, and treatment response of solid cancers. Biologically, elevated stiffness promotes proliferation (Ulrich et al., 2009), invasiveness (Tse et al., 2012), and metastasis (Wirtz et al., 2011) through activation of mechanosensitive signaling pathways; clinically, an increase in stiffness is associated with an increased risk of breast cancer (Boyd et al., 2014) and mortality (Evans et al., 2012). In diagnostics, cellular and extracellular stiffness are traditional markers of cancer (Cochlin et al., 2002; Goenezen et al., 2012) and are predictive of a tumor’s stage (Panzetta et al., 2017). In therapy, increased stiffness is linked to reduced efficiency of drug delivery (Nia et al., 2019). Furthermore, determining the stiffness of the tumors and their surrounding tissue is an essential precursor for estimating solid mechanical stresses, another physical hallmark of cancer (Nia et al., 2016; Nia et al., 2018; Nia et al., 2019; Nia et al., 2020b; Zhang et al., 2023a; Zhang et al., 2023b). Despite this, the lung’s or a lung tumor’s stiffness has not been reported under the following conditions: (i) across a range of physiologically relevant pressures, (ii) noninvasively, i.e., without sectioning of the tissue, (iii) under realistic boundary conditions, and (iv) at microscale resolution.

Although elastography encompasses a broad range of techniques for assessing the material properties of biological tissues, each method presents limitations when addressing our specific problem (Costa, 2004; De et al., 2010; Evans et al., 2012; Goenezen et al., 2012; Kennedy et al., 2014; Garra, 2015; Schregel et al., 2018; Seidl et al., 2020; Silva et al., 2020; Ghiuchici et al., 2021; Kuo et al., 2021; Wang et al., 2023). The gold-standard method for microscale elastography is atomic-force microscopy (Krieg et al., 2018), which boasts extremely high-resolution, absolute measurements of stiffness. However, tissue preparation for AFM involves resection and submersion in saline, which disrupts the mechanical integrity of the sample and the alveolar air-liquid interface (Liu and Tschumperlin, 2011). Though CT and MRI elastography preserve the mechanical environment of the organ, these methods have poor spatiotemporal resolution, and they typically report strain rather than absolute stiffness (Barbone and Bamber, 2002; Barbone and Gokhale, 2004). Although strain elastography based on modalities like synchrotron microCT (Cercos-Pita et al., 2022) have near-micron spatial resolution, to our knowledge, these methods lack the control and temporal resolution needed for tracking the same region of interest at alveolar resolution across changes in inflation pressure. Optical elastography (Kennedy et al., 2014; Kennedy et al., 2017; Wang et al., 2023) offers an alternative method for more precisely estimating the displacements throughout a biological sample. For example, recent papers have implemented optical elastography based on digital image correlation (DIC) to quantify the lung’s strain (Nelson et al., 2023) and stiffness (Maghsoudi-Ganjeh et al., 2021); but in each case, the empirical method does not provide physiologically realistic boundary conditions, and the measurements are not at alveolar resolution. Using optical elastography based on deformable image registration, our group recently mapped the elasticity of resected biological samples at optical resolution either by embedding them in thermo-responsive hydrogels (Regan et al., 2023) or by adhering precision-cut lung slices (Kim et al., 2023). However, these methods also involve resection of the organ and embedding the sample in saline, which does not preserve the organ’s boundary conditions and disrupts the air-liquid interface in the lung.

With that goal in mind, we recently developed the crystal ribcage (Banerji et al., 2023), which preserves the integrity of the organ and emulates the *in vivo* boundary conditions seen by the lung, while at the same time enabling real-time microscopy of the entire surface during dynamic ventilation at cellular resolution (Supplementary Figure S1). Unlike intravital imaging methods (Looney et al., 2011; Looney and Bhattacharya, 2014; Entenberg et al., 2018) wherein the lung is immobilized by vacuum or glue, and which thus compromise the breathing mechanics of the lung at the imaging site, the plasma-treated crystal ribcage provides a lubricious, geometrically realistic boundary condition, allowing mechanical characterization of the lung throughout the breathing cycle in health and disease. While the tissue preparation involves resection of the organ from the mouse’s thorax, the *ex vivo* lung, with its pleura intact, is imaged immediately after resection, and the lung can be vascularly perfused with complete media to maintain cell health throughout the course of imaging. Consequently, our platform preserves the *in vivo* physiological conditions of the lung. By developing an optical elastography platform based on the crystal ribcage apparatus, we can assess the mechanical properties of the *ex vivo* lung in health and disease with high spatiotemporal resolution and with physiologically realistic boundary conditions.

Here, to accurately estimate the *in vivo* mechanical properties of the lung in health and disease, we adopt a multiscale-modeling approach that couples the microscale displacements estimated through deformable image registration and the mean, strain-dependent stiffnesses estimated using a nonlinear, finite-element model of the lung. We validate the multiscale model against a virtual, finite-element model of the lung with a cancerous tumor, demonstrating that the method is capable of accurately recovering the mechanical properties throughout the domain even in the presence of pathology. Upon applying the model to images of the lung within the crystal ribcage, we find that (i) the stiffness of the lung tissue increases nonlinearly with transpulmonary pressure across the full range of end-expiratory to end-inspiratory pressures; (ii) there is significant heterogeneity in material properties at alveolar resolution; (iii) the intratumor stiffness increasingly exceeds the extratumor stiffness across the entire range of pressures; and finally, (iv) the variance in stiffness increases with strain for both the healthy and cancerous tissue. While the present study characterizes the micromechanics of the healthy lung and the lung with cancerous tumors, the method has the potential to be applied to a wide range of disease states such as fibrosis, COPD, and respiratory infections.

## 2 Methods

### 2.1 Mouse model of lung cancer, crystal ribcage fabrication, and imaging

#### 2.1.1 Animal use ethics

All experiments conformed to the ethical principles and guidelines under protocols set forth and approved by the Boston University Institutional Animal Care and Use Committee (protocol number PROTO201900086). All animal procedures were compliant with ARRIVE guidelines. Mice were housed in ambient temperature and humidity and 12-h light–dark conditions under pathogen-free conditions at the Boston University Animal Science Center. No housing or handling exceptions were made for this study.

#### 2.1.2 Mice

We used 11- to 23-week-old male and female mice for experimental procedures including healthy lung imaging and generating models of primary cancer, as previously described (Banerji et al., 2023). A breeding pair of transgenic B6.129(Cg)-Gt (ROSA)26Sortm4 (ACTB-tdTomato,-EGFP)Luo/J mice (JAX, 007676, Jackson Labs) (Muzumdar et al., 2007), referred to by the abbreviation “mTmG”, was initially purchased to breed a colony; that colony was the source of all animals for healthy lung and primary cancer experiments. For the present study, which examines two representative mice from this colony, the healthy mouse was 11 weeks old at the time of imaging. The urethane mouse, serving as our model of primary cancer, was 23 weeks at the start of urethane dosing and 54 weeks at the time of imaging. Between these ages, the murine lung’s volume does not change appreciably both in our experience and per development studies (Schulte et al., 2019).

#### 2.1.3 Primary cancer model

We adapted a previously described protocol (Janker et al., 2018; Sozio et al., 2021) to induce primary lung cancer in mTmG mouse lungs using urethane (Sigma U2500). A stock solution of urethane was prepared at a working concentration of 200 mg/mL in PBS. Mice were dosed with the urethane solution at 1 mg/g body weight, twice weekly for 5 weeks by intraperitoneal (IP) injection. Mice were sacrificed and lungs harvested for imaging in the crystal ribcage after 6–12 months. The maximum tumor size permitted for the study was 1.5 mm in diameter. Mice were excluded from the study after presenting with labored breathing, hunched posture, or ruffled fur due to tumor progression.

#### 2.1.4 Crystal ribcage fabrication

The full development of the crystal ribcage platform is described in our previous work (Banerji et al., 2023). Briefly, microCT scans of C57BL/6 mouse chest cavity (courtesy the Hoffman group at the University of Iowa (Thiesse et al., 2010; Vasilescu et al., 2012; Kizhakke Puliyakote et al., 2016)) were segmented and refined to create the native ribcage geometry. In successive additive manufacturing and fabrication steps the ribcage model was converted into the crystal ribcage mold that was thermoformed over to create the polystyrene crystal ribcage. The internal surface was engineered to be hydrophilic to allow the lung to glide over its surface, as in the native ribcage. A six degree of freedom arm was included to rotate the crystal ribcage about any axis to image across the entire the distal lung surface using either a top-down or bottom-up configured microscope. Because lung volume changes significantly with age (Schulte et al., 2019), we have fabricated different, age-specific crystal ribcages to accommodate lungs of different sizes (Banerji et al., 2023).

#### 2.1.5 Lung preparation

Isolated mouse lungs were ventilated and perfused as previously described (Vanderpool and Chesler, 2011; Banerji et al., 2023). Briefly, the mouse trachea was cannulated and the lungs dynamically ventilated (Kent Physiosuite Mouse Ventilator, Kent Scientific). The lungs were perfused by cannulating the pulmonary artery and left atrium, and perfusing serum-free RPMI cell culture medium (Corning) through the lung vasculature. After cannulating the trachea and mouse heart, the lung–heart bloc, was excised and placed into the crystal ribcage for *ex vivo* microscopy under variable quasi-static positive air pressures.

#### 2.1.6 Lung microscopy

As previously described (Banerji et al., 2023), *ex vivo* lungs, under quasi-static inflation conditions and within the crystal ribcage, were imaged using (i) an upright Nikon stereomicroscope with a 1x objective, and (ii) an upright Nikon CSU-X1 spinning-disk confocal microscope with 1x, 2x, 4x and 10x objectives, using NIS-Elements acquisition software and with the environmental temperature control set to 37°C.

Z-stacks of the diseased and healthy lungs were acquired on the confocal microscope using a 561 nm laser at 20–50 ms exposure (50–20 frames per second) per frame. Voxel sizes varied based on objective used, with XY resolution varying from 1–10 μm and Z step sizes varying from 2.5–12.5 μm. Total Z-stack acquisition time was on the order of 6–15 s for each positive-end expiratory pressure (PEEP) condition.

Before imaging, lungs were gradually recruited by slowly raising the intratracheal pressure to 18 cmH_2_O, measured using custom sensors sensitive to 0.1 cmH_2_O, using a water column. The pressure was then reduced in decrements of 1 cmH_2_O down to 2 cmH_2_O. To allow the lung to relax to its steady-state condition, each pressure was maintained for 1 min before imaging.

### 2.2 Organ-scale geometry modeling

**FIGURE 1:**
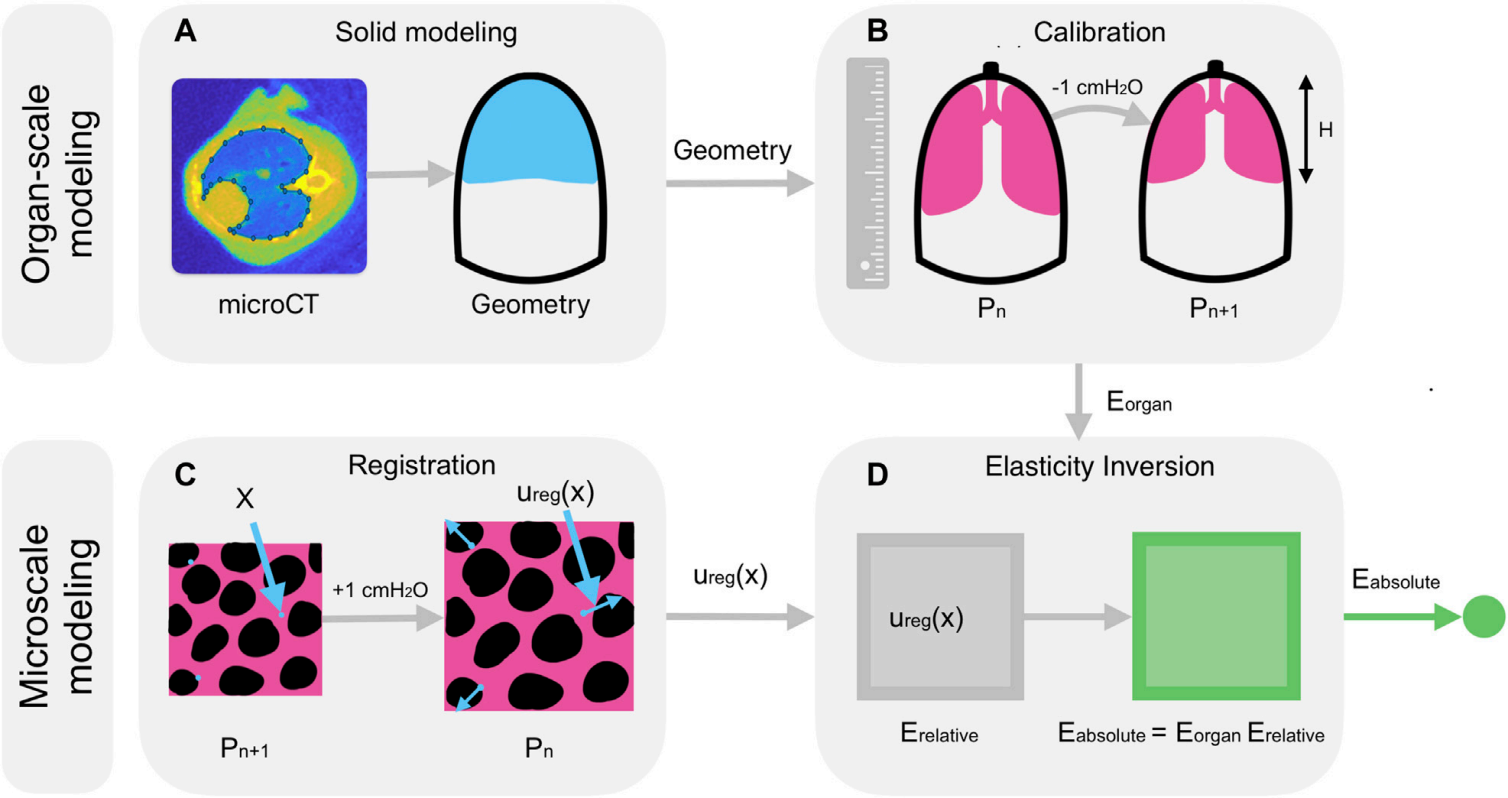
Model description. **(A)** A Bayesian classifier segments the geometries of the lung and of the ribcage from microCT images (Thiesse et al., 2010; Vasilescu et al., 2012; Kizhakke Puliyakote et al., 2016) of the mouse thorax, and the segmentation is then used to construct a solid geometry in SolidWorks for finite-element analysis. **(B)** After recruitment, the height, H , of the organ in the crystal ribcage is measured as the distending transpulmonary pressure decreases from 18 to 2 cmH_2_O in decrements of 1 cmH_2_O, with the pressure at the start of the *n*th step denoted by $P_{n}$ , and the material constants of the finite-element model are chosen to reproduce this pressure-distension curve. **(C)** Deformable image registration determines how material points, x , within the lung tissue displace with microscale resolution in response to an increment in pressure of 1 cmH_2_O, producing the estimated displacements, $u_{reg}\left(x\right)$ , for that step, and **(D)** the inverse elasticity problem is solved using an implementation of the Adjoint-Weighted Equation formulation (Barbone et al., 2010) of the inverse elasticity problem in Fenics (DOLFINx, 2023), an open-source Python framework for numerically solving differential equations in scientific computing applications. From the measured displacements, we recover an estimate of the elastic modulus in relative units, $E_{\text{relative}}$ , having unity mean. Upon rescaling this estimate so that its mean value matches that of the organ-scale model, $E_{\text{organ}}$ , we recover the absolute stiffness throughout the microscale domain, $E_{\text{absolute}}$ .

Figure 1 summarizes the multiscale model. In short, we (i) segment microCT images of the lung in MATLAB 2022b (The MathWorks, Inc.), (ii) construct a geometric model from the segmentation in SolidWorks 2021 (Dassault Systèmes), (iii) simulate ventilation of the organ-scale model in Abaqus 2022 (Dassault Systèmes) for a range of material coefficients, (iv) determine the coefficients that optimally reproduce the observed pressure-distension behavior of the lung in the crystal ribcage, (v) solve the inverse elasticity problem for the distribution of material properties throughout the microscale domain in arbitrary units, and finally, (vi) rescale these relative stiffnesses so that the mean value matches the stiffness of the organ-scale model, yielding our goal of recovering the absolute stiffnesses throughout the microscale domain. Given a three-dimensional microCT image (Thiesse et al., 2010; Vasilescu et al., 2012; Kizhakke Puliyakote et al., 2016) of the mouse thorax, we first construct three-dimensional geometric models (Figure 1A) of the mouse lung and ribcage for finite-element analysis as follows.

#### 2.2.1 Segmenting the lung and ribcage

Due to significant variations in the lung’s intensity within a microCT volume, segmenting the organ by thresholding is unreliable. To segment the lung, we thus construct a naïve, Bayesian classifier—trained on a single, two-dimensional slice of the image along with its ground-truth class labels—to differentiate the lung class L from its surroundings M (Bishop, 2006). Let $\Omega \subset R^{3}$ be the position vectors of pixels within the domain of the microCT image (Thiesse et al., 2010; Vasilescu et al., 2012; Kizhakke Puliyakote et al., 2016), so that $\vec{x}\in \Omega$ is the position of a given voxel. The image can then be expressed as the scalar field $I\left(\vec{x}\right):\Omega \to R$ . Let $F\left(\vec{x}\right):\Omega \to R^{n}$ map from each of these position vectors to the n-dimensional feature vectors extracted from $I\left(\vec{x}\right):\Omega \to R$ . In the present study, each feature vector has ten components, $\varphi_{i}\left(\vec{x}\right):\Omega \to R$ , each corresponding to a transformation of the image volume by a different neighborhood operation. These components are listed in Table 1.

**TABLE 1:** Components of the feature vectors extracted from the microCT volume for building the Bayesian classifier. Each feature vector has ten components, each corresponding to a different transformation of the image volume. To normalize the components, each component is divided by its standard deviation.

Feature-vector component	Description
$\varphi_{1}$	Sobel filter with threshold 0.1 applied to I
$\varphi_{2}$	Sobel filter with threshold 0.4 applied to I
$\varphi_{3}$	Gaussian filter of variance 2 applied to $\varphi_{1}$
$\varphi_{4}$	Gaussian filter of variance 4 applied to $\varphi_{2}$
$\varphi_{5}$	Laplacian-of-Gaussian filter applied to I
$\varphi_{6}$	Gaussian filter of radius 4 applied to I
$\varphi_{7}$	Gaussian filter of radius 8 applied to I
$\varphi_{8}$	Gaussian filter of radius 16 applied to I
$\varphi_{9}$	The original image I
$\varphi_{10}$	Morphological dilation of $\varphi_{2}$

Let $\Omega_{L}\subset \Omega$ be the subset of position vectors from a given slice of the image volume that have been manually labeled as belonging to the lung, and let $\Omega_{M}\subset \Omega$ be the remaining position vectors from the same slice. For the priors, we assume that the prior probability $P\left(L\right)=\frac{\left|\Omega_{L}\right|}{\left.\left|\Omega_{L}\right|\left.+\right|\Omega_{M}\right|}$ , which implies by the axiom of normalization that $P\left(M\right)=1-P\left(L\right)$ . To estimate the likelihoods, $P\left(\vec{x}\;|\;L\right)$ and $P\left(\vec{x}\;|\;M\right)$ , we train Gaussian mixture models on the feature vectors $F\left(\Omega_{L}\right)$ and $F\left(\Omega_{M}\right)$ , respectively. Finally, from these definitions, we apply Bayes’ theorem to recover $P\left(\left.L\;\right|\;\vec{x}\right)$ , the posterior probability that a given pixel corresponds to lung tissue.

$$P\left(\left.L\;\right|\;\vec{x}\right)=\frac{P\left(\left.\vec{x}\;\right|\;L\right)P\left(L\right)}{P\left(\left.\vec{x}\;\right|\;L\right)P\left(L\right)+P\left(\left.\vec{x}\;\right|\;M\right)P\left(M\right)}$$ (1)

The lung segmentation $S_{L}\left(\vec{x}\right):\Omega \to \left\{0,1\right\}$ is then defined as $S_{L}\left(\vec{x}\right)=1$ if $P\left(\left.L\;\right|\;\vec{x}\right)>0.5$ and $S_{L}\left(\vec{x}\right)=0$ otherwise. In the MATLAB implementation, these maps— ${I\left(\vec{x}\right),F\left(\vec{x}\right),S}_{L}\left(\vec{x}\right),$ and $P\left(\left.L\;\right|\;\vec{x}\right)$ —are represented as matrices.

In contrast, because the intensity of bone tissue is much higher than that of other biological materials, the approach to segmenting the ribcage is simpler. Here, the ribcage segmentation $S_{R}\left(\vec{x}\right):\Omega \to \left\{0,1\right\}$ is defined as $S_{R}\left(\vec{x}\right)=1$ if and only if the intensity $I\left(\vec{x}\right)$ exceeds some constant, volume-dependent threshold. For both the lung segmentation and the ribcage segmentation, the resulting segmentation contains multiple connected components, corresponding to features like the scapula, humerus, cartilaginous tracheal rings, and tissue outside of the lung; extracting the largest connected components from these initial segmentations isolates the desired region of interest.

#### 2.2.2 Constructing solid models of the lung and ribcage

From the lung segmentation $S_{L}\left(\vec{x}\right)$ , we approximate the surface of the diaphragm as follows. First, we find $z_{\max}\left(x,y\right)=\max\;\left.\left\{\;\right.z\;\right|\;\left(x,y,z\right)\in \text{Ω}⋀S_{L}\left(x,y,z\right)\left.=1\right\}$ . We then filter the mapping $z_{\max}\left(x,y\right)$ using a mode filter. Finally, we resample points from this surface using a thin-plate smoothing spline and save the point cloud to a text file (Hastie et al., 2009). This point cloud represents the geometry of the diaphragm.

Next, to construct a point-cloud approximation of the ribcage, we first find the geometric centroid of the lung, ${\vec{x}}_{c}$ , from $S_{L}\left(\vec{x}\right)$ using the following equation.

$${\vec{x}}_{c}=\frac{1}{\sum_{\vec{x}\in \Omega }S_{L}\left(\vec{x}\right)}\sum_{\vec{x}\in \Omega }{\vec{x}\;S}_{L}\left(\vec{x}\right)$$ (2)

For each slice of the volume, we then project rays from the projection of the centroid, ${\vec{x}}_{c}$ , onto the given slice at a dense collection of angles from 0 to 2π until each ray contacts a nonzero pixel on the interior of the ribcage segmentation $S_{R}\left(\vec{x}\right)$ . A thin-plate smoothing spline is then fit to these contact points to produce a surface approximating the ribcage, and a dense collection of points, $P_{R}$ , are sampled from this surface. Because the ribcage is open near the apex of the lung, these sampled points are artifactually peaked in the neighborhood of the apex. To correct this, the nodes near the apex are flattened by minimizing the following objective function.

$$\sum_{i}{\left(k\left(z_{i}-Z_{i}\right)-g_{z}\right)}^{2}+\alpha {\left(\nabla_{xy}z_{i}\right)}^{2}+\beta {\left(\nabla_{xy}^{2}z_{i}\right)}^{2}+\gamma {\left(\nabla_{xy}\left(z_{i}-Z_{i}\right)\right)}^{2}+\delta {\left(\nabla_{xy}^{2}\left(z_{i}-Z_{i}\right)\right)}^{2}$$ (3)

In this equation, $z_{i}$ represents the z-component of the *i*th node’s position vector after correction, $Z_{i}$ represents the same component before correction, k represents the stiffness of a virtual spring anchoring a point to its original height, and $g_{z}$ is a body force pulling these points toward the centroid of the ribcage. The remaining terms serve to regularize the optimization, penalizing the first and second derivatives of the height as well as changes in these derivatives. The sum is taken over the points near the apex of the lung. Finally, these point clouds of the diaphragm and ribcage are saved as text files for subsequent import into SolidWorks.

From these point clouds, we finally construct STEP (Standard for the Exchange of Product model data defined by ISO 10303 (Pratt, 2001)) representations of the diaphragm and the ribcage using the ScanTo3D feature in SolidWorks. By cutting the ribcage surface with the diaphragm surface, and filling the space enclosed between them, we recover a simplified model of the lung that is everywhere tangent to the ribcage. This approach guarantees *a priori* that, at the start of each simulation, the lung geometry and the ribcage geometry are in perfect contact, preventing errors in predicted strains and stresses that may arise due to mismatch between these geometries. The STEP representations of the ribcage and the lung are then exported from SolidWorks.

### 2.3 Organ-scale finite-element modeling

#### 2.3.1 Simulating the healthy lung

To perform finite-element simulations of the organ, these STEP geometries are now imported into Abaqus 2022 (Dassault Systèmes). The ribcage is taken to be a discrete, rigid part and is meshed with rigid, triangular elements. The lung is taken to be a deformable part and is meshed with C3D10 quadratic tetrahedral elements, which are chosen over C3D4 linear tetrahedral elements for their tendency to converge more quickly with coarser mesh resolutions. In simulations of the healthy lung, the lung mesh consists of 34,541 nodes and 21,945 elements; the ribcage mesh consists of 64,093 nodes and 127,697 elements. The simulation was performed on the Boston University Shared Computing Cluster hosted by the Massachusetts Green High-Performance Computing Center distributing the load over 12 processors with 4 GB of RAM per processor, each simulation completed within 7 h.

Based on prior studies and on the lung’s microstructure and constitutive behavior—which resembles a hyperelastic, tetrakaidekahedral foam whose walls are comprised of elastin, type-I collagen, and type-III collagen—the lung is modeled using the Ogden-Hill model (Berezvai and Kossa, 2017) of a hyperelastic foam (Vawter et al., 1979; Andrikakou et al., 2016). The general form of the strain-energy density function is thus taken to be

$$U=\sum_{i=1}^{N}\left(2\mu_{i}/\alpha_{i}^{2}\right)\left(\lambda_{1}^{\alpha_{i}}+\lambda_{2}^{\alpha_{i}}+\lambda_{3}^{\alpha_{i}}-3+\frac{1}{\beta_{i}}\left(J^{-\alpha_{i}\beta_{i}}-1\right)\right),$$ (4)

where $\alpha_{i}$ is a dimensionless material parameter determining the nonlinear behavior of the stress-strain relation, $\beta_{i}$ is a dimensionless material parameter given by the Poisson’s ratio as $\beta_{i}=\nu_{i}/\left(1-2\nu_{i}\right)$ , $\mu_{i}$ is a material parameter with units of stress determining the shear modulus during small strains from the reference configuration, $\lambda_{i}$ is the $i^{th}$ principal stretch, J is the determinant of the deformation gradient, and N is the number of terms in the model. For simplicity, we assume that the strain-energy density function consists of only one term, reducing the general equation to

$$U=\left(2\mu /\alpha^{2}\right)\left(\lambda_{1}^{\alpha }+\lambda_{2}^{\alpha }+\lambda_{3}^{\alpha }-3+\frac{1}{\beta }\left(J^{-\alpha \beta }-1\right)\right).$$ (5)

This strain-energy density function, and consequently the pressure, is linear in the parameter μ and nonlinear in the parameter α . Therefore, if we simulate the distensions for parameters ( $\left.\mu ,\alpha \right)$ , then the pressures required to produce the same distensions for any other $\left(\mu^{*},\alpha \right)$ are a simple rescaling of those for ( $\left.\mu ,\alpha \right)$ . In practice, therefore, it is unnecessary to simulate ventilation for the same α but different μ to determine the pressure-distension curve.

The boundary conditions (Supplementary Figure S2) include immobilization of the ribcage, frictionless sliding contact between the lung’s upper surface and the ribcage, and negative pressure on the boundary of the lung. Although experiments involve positive-pressure ventilation, the simulation involves applying negative pressure to the external surface of the lung; the explanation for this apparent discrepancy is that the governing equations are symmetric under mutual inversion of the pressure’s sign and the surface normal’s direction, implying that the model is equally applicable to either mode of ventilation (Shi et al., 2022). Since the parts have been designed *a priori* to be tangent everywhere, initial contact between the surfaces is easy to establish.

While previous studies (Tawhai et al., 2009; Shi et al., 2022) have shown that gravity significantly influences the mechanics of the human lung, our model neglects the influence of gravity due to its smaller role in the mouse. Consider the conservation of linear momentum under conditions of static equilibrium, which has been rendered dimensionless (Munson et al., 2012) by factoring out the lung density $\rho_{\text{lung}}$ , gravitational acceleration g , lung height $H_{\text{lung}}$ , and transpulmonary pressure $P_{\text{tp}}$ .

$$\frac{P_{\text{tp}}}{\rho_{\text{lung}}gH_{\text{lung}}}\nabla^{*}\cdot P^{*}+B^{*}=0$$ (6)

Here, $\nabla^{*}$ , $P^{*}$ , and $B^{*}$ represent the dimensionless divergence operator, the dimensionless tissue stress, and the dimensionless gravitational body forces, respectively. The tissue stress is known to be approximately equal to the transpulmonary pressure (Mead et al., 1970). Consequently, the dimensionless number $\frac{P_{\text{tp}}}{\rho_{\text{lung}}gH_{\text{lung}}}$ characterizes the magnitude of the tissue stress divergence relative to the magnitude of gravity. In the human lung, this dimensionless quantity remains below 1 for transpulmonary pressures up to 20 cmH_2_O, indicating the significant role of gravity in governing its mechanics. In the mouse lung, however, this dimensionless quantity is equal to 1 when the transpulmonary pressure is 1 cmH_2_O, but decreases linearly as the transpulmonary pressure increases; once the transpulmonary pressure reaches 10 cmH_2_O, this dimensionless number increases to 10, indicating that tensile forces within the tissue greatly exceed gravitational body forces. Based on this reasoning, we posit that it is reasonable to neglect gravity when modeling the murine lung, with the approximation improving at higher pressures. Additional reasoning is discussed in the results.

Finally, it is important to note that the lung exhibits hysteresis, with its inflation characterized by one strain-energy density function and its deflation characterized by another (Vawter et al., 1979). In this study, we elect to model the lung’s behavior during quasistatic deflation, so that our measurements used to calibrate the model are taken from states of higher pressure to states of lower pressure in near-equilibrium. The same approach can easily be repeated to recover a model of the lung’s behavior during inflation.

#### 2.3.2 Simulating the cancerous lung

To simulate the cancerous lung, the same process is repeated, but rather than being constant, the material field $\mu \left(\vec{X}\right)$ is defined as

$$\mu \left(\vec{X}\right)={Ae}^{-{\left(\vec{X}-{\vec{X}}_{\text{tumor}}\right)}^{n}/\sigma^{n}}+B,$$ (7)

where ${\vec{X}}_{\text{tumor}}$ is the centroid of the spherical tumor in material coordinates, σ determines its width, n controls the shape of the decay in magnitude with distance, and the coefficients A and B determine the stiffness in the near and far fields. To illustrate, we note that $\mu \left(\vec{X}\right)$ approaches A+B as $\vec{X}$ approaches ${\vec{X}}_{\text{tumor}}$ , and that $\mu \left(\vec{X}\right)$ approaches B as $\left\|\vec{X}-{\vec{X}}_{\text{tumor}}\right\|$ approaches infinity. Consequently, A+B is the stiffness at the tumor’s centroid, while B is the stiffness far from the tumor. This material equation possesses three attractive properties: (i) it is spherically symmetric, corresponding to our interpretation that the equation represents a spherical tumor, (ii) stiffness decays with distance from the centroid, reflecting our intent that the tumor is stiffer than its surroundings, and (iii) it is differentiable and smooth, making it easier to work with during numerical computations. When n=2 , the field is a multidimensional normal distribution, and as n→∞ , the field approaches an indicator function for a ball. In our studies, we chose n=4 .

The ribcage mesh is the same as before, while the lung mesh now consists of 12,679 nodes and 7,766 elements. Adaptive mesh refinement, which was necessary for convergence in the neighborhood of the tumor, yielded a mesh with fewer elements relative to the simulation of the healthy lung. The simulation was performed on the Boston University Shared Computing Cluster hosted by the Massachusetts Green High-Performance Computing Center; distributing the load over 12 processors with 4 GB of RAM per processor, the simulation completed within 4 h.

#### 2.3.3 Calibrating the organ-scale finite-element model

**FIGURE 2:**
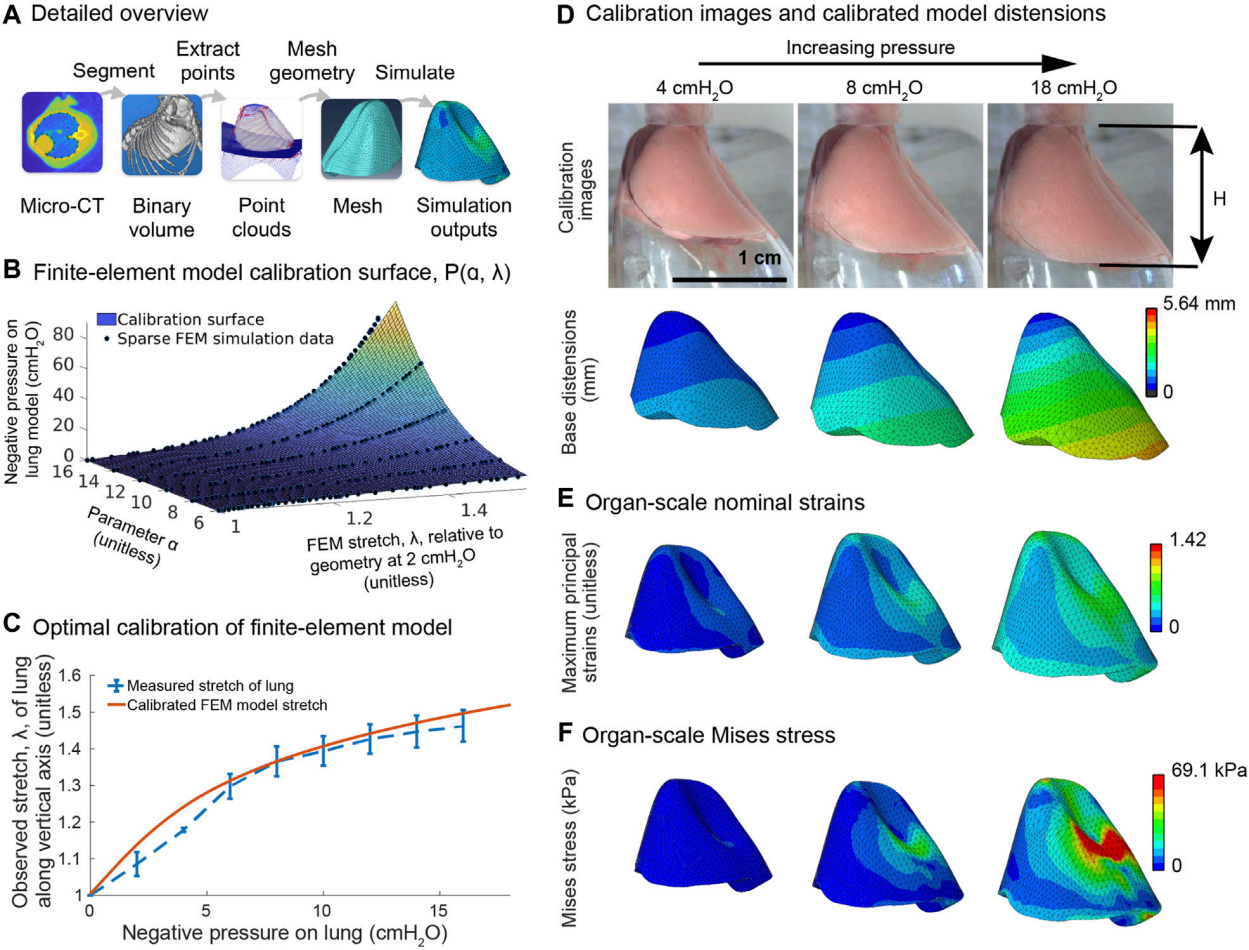
Constructing the finite-element model of the lung from a microCT volume and organ-scale pressure-distension data. **(A)** A Bayesian classifier segments the mouse lung and ribcage from a microCT volume (Thiesse et al., 2010; Vasilescu et al., 2012; Kizhakke Puliyakote et al., 2016) of the mouse thorax. From the segmentations, we construct smooth point clouds approximating the interior surfaces of the ribcage and of the diaphragm. From these point clouds, we construct solid models in SolidWorks and then mesh those models in Abaqus. **(B)** Pressure *versus* vertical stretch, λ, is recorded for simulations across a range of values for the material parameter α . Because the model is linear in the material parameter μ , this allows us to produce a predictive model of transpulmonary pressure *versus* distension for a wide range of material coefficients based on a limited number of forward simulations. The parameter α controls the nonlinearity of the model. The parameter μ controls the initial slope of the model in the small-strain regime. Having characterized the model in **(B)**, we can solve **(C)** for the optimal material coefficients from stereomicroscope images of the lung in the crystal ribcage shown in **(D)**. **(E, F)** Strains and stresses in the lung throughout the finite-element model.

The finite-element simulation is repeated for different material parameters within a neighborhood of the values yielding optimal agreement between empirical and *in silico* outcomes. When the strain-energy density function is restricted to a single term, the parameter μ represents the material’s shear modulus during small strains, as can be shown by a Taylor expansion of the strain-energy density function about λ=1 . From the stress-strain curve in the linear regime, when the strain increases linearly with distending pressure, we can estimate the Young’s modulus as the ratio of the increment in strain to the increment in pressure. From Figure 2C, we estimate that incrementing the pressure from 0 kPa to 0.5 kPa yields an increment in strain of 0.2. Following this reasoning, we find that E≈0.5 kPa/0.2=2.5 kPa . Assuming a Poisson’s ratio of ν=0.2 (Concha et al., 2018), this value for the Young’s modulus corresponds to a shear modulus of $\mu =E/\left(2\left(1+\nu \right)\right)\approx 1\;kPa$ .

Based on these initial estimates, we perform simulations for μ=1 kPa and the dimensionless parameter α ranging from six to 16. The outcomes of these simulations are then used to construct a calibration surface $P\left(\lambda_{H},\mu ,\alpha \right)$ , where $\lambda_{H}$ is the stretch of the finite-element model along the vertical axis, μ and α are the material parameters mentioned earlier, and P is the transpulmonary pressure required to distend the finite-element model to this degree of stretch. From the observed distensions of the lung in the crystal ribcage (Figure 1B) for pressures ranging from 2 to 18 cmH_2_O, $\left(\lambda_{obs},P_{obs}\right)$ , we then determine the maximum likelihood assignments for μ and α by minimizing the following objective function (Bishop, 2006).

$$\sum_{\left(\lambda_{obs},P_{obs}\right)}{\left(P\left(\lambda_{obs},\mu ,\alpha \right)-P_{obs}\right)}^{2}$$ (8)

This optimization procedure is not computationally intensive; running on a personal laptop with 16 GB of RAM and a typical CPU, for example, the process completes within seconds. The outcome is a single, point estimate of the coefficients that characterize the organ-scale behavior. It should be noted that, because the model is linear in the parameter μ , we can in practice simplify the problem of determining the optimal parameters by transforming the objective function as follows.

$$\sum_{\left(\lambda_{obs},P_{obs}\right)}{\left(\mu P\left(\lambda_{obs},1,\alpha \right)-P_{obs}\right)}^{2}$$ (9)

This implies that, rather than sampling the function $P\left(\lambda_{obs},\mu ,\alpha \right)$ , we only need to sample the function $P\left(\lambda_{obs},1,\alpha \right)$ in our finite-element simulations. In contrast, because the model is nonlinear in α , an analogous transformation is not possible for α ; this is why we limit the constitutive model to a single term.

### 2.4 Inverse elasticity problem at the microscale

#### 2.4.1 Measuring the displacements using image registration

To measure the displacements caused by a change in distending pressure applied to the lung at cellular resolution, we leverage deformable image registration (Heinrich et al., 2012a) (Figure 1C). To prepare the images for registration, we perform an optimization to autonomously correct the bulk rotation of the material due to the natural curvature of the crystal ribcage away from the imaging plane. Because the image-registration algorithm does not, in practice, yield good estimates for the displacements along the shallow depth axis, we project the rotationally corrected images along this axis.

After preprocessing the images, we invoke the image-registration algorithm. This algorithm, which has been adapted from earlier literature (Heinrich et al., 2012a), formulates the inverse-elasticity problem as an inference problem on a Markov Random Field (MRF) to automatically determine the displacements necessary to match images of the lung at two different pressures. The observable variables of the Markov Random Field are modality-independent neighborhood descriptors (MIND) extracted from the image (Heinrich et al., 2012b), while the latent variables are the displacements necessary to minimize the sum of squared differences in these descriptors across the two images; edges between the latent variables of neighboring observables represent the constraint that displacements vary smoothly throughout the domain. Let O and T be the original and deformed images, respectively. Furthermore, let $W\left(O,u\right)$ be the image produced by warping the image O using the displacements u . Lastly, let the function D map an image to its MIND representation. Then the registration algorithm finds the displacements that minimize the objective function

$$\sum_{\vec{x}\in \Omega }{\left(D\left(W\left(O,u\right)\right)-\;D\left(T\right)\right)}^{2},$$ (10)

where the sum is taken over the points in the image domain, Ω . From these displacements and the material properties of the calibrated finite-element model, we next find the distribution of stiffnesses throughout the domain, as described in the next section.

#### 2.4.2 Solving for the shear modulus parameter

To determine the relative stiffnesses of the material throughout the image domain, we leverage a Python implementation of the Adjoint-Weighted Equation (AWE) formulation of the inverse-elasticity problem (Barbone et al., 2010). Let $\vec{X}\in \Omega_{0}\subset R^{2}$ be the coordinates of material points of a deformable body in the reference configuration, and let $\vec{x}\in \Omega_{1}\subset R^{2}$ be the coordinates of the same material points after some deformation. Because soft tissues deform continuously, there exists a continuous function $\psi :R^{2}\to R^{2}$ such that $\vec{x}=\psi \left(\vec{X}\right)$ . Given the registered displacements, $u\left(\vec{X}\right)=\psi \left(\vec{X}\right)-\vec{X}$ , along with the definition of the deformation gradient, $F\left(\vec{X}\right)=\nabla_{\vec{X}}\psi \left(\vec{X}\right)$ , we can recover the deformation gradient tensor in terms of $\vec{X}$ (Holzapfel, 2000).

$$F\left(\vec{X}\right)=I+\nabla_{\vec{X}}u\left(\vec{X}\right)$$ (11)

From the deformation gradient, we next determine the Cauchy-Green strain tensor field, $C\left(\vec{X}\right)$ , as follows.

$$C\left(\vec{X}\right)={F\left(\vec{X}\right)}^{T}F\left(\vec{X}\right)$$ (12)

By the spectral theorem and the manifest symmetry of $C\left(\vec{X}\right)$ , we subsequently determine the eigenvalues, ${\lambda_{1}\left(\vec{X}\right)}^{2}$ and ${\lambda_{2}\left(\vec{X}\right)}^{2}$ , of this tensor.

The areal strain, which is a scalar field representing the fractional change in the material’s area relative to its value in the reference configuration, is then given by

$$\varepsilon_{A}\left(\vec{X}\right)=\lambda_{1}\left(\vec{X}\right)\lambda_{2}\left(\vec{X}\right)-1.$$ (13)

The whole organ’s stress-strain behavior is well-described by a hyperelastic material law, where the nominal stress is the derivative of the strain-energy function with respect to the principal nominal stretches. If we assume that this model holds at all length scales, down to the cellular scale and for both healthy and diseased tissue, then we can use this same constitutive model when solving the inverse problem. Therefore, from the same strain-energy function introduced earlier, we recover the principal components of the first Piola-Kirchhoff stress tensor as follows.

$$P_{i}=\frac{\partial W}{\partial \lambda_{i}}=\mu \left(\vec{X}\right)\left(\frac{2}{\alpha \lambda_{i}}\left(\lambda_{i}^{\alpha }-J^{-\alpha \beta }\right)\right)$$ (14)

where $\beta =\frac{\nu }{1-2\nu }$ . In evaluating the above expression, we need to compute the Jacobian determinant $J=\lambda_{1}\lambda_{2}\lambda_{3}$ , but image registration only yields the stretches tangential to the lung’s surface. Consequently, we approximate J as $J\approx {\left(\lambda_{1}\lambda_{2}\right)}^{3/2}$ . Finite-element simulations of the whole organ indicate that this approximation generally holds within 10%–15% error (Supplementary Figure S2).

For a hyperelastic material, it is well-known that the eigenvectors of the stress are aligned with the eigenvectors of the strain. Therefore, if ${\vec{v}}_{i}$ are the principal directions of the right Cauchy-Green strain tensor, the first Piola-Kirchhoff stress tensor becomes

$${P=P}_{1}{\vec{v}}_{1}⊗{\vec{v}}_{1}+P_{2}{\vec{v}}_{2}⊗{\vec{v}}_{2}.$$ (15)

We can simplify the above expression by defining the tensor $A\left(\vec{X}\right)=P\left(\vec{X}\right)/\mu \left(\vec{X}\right)$ , leading to the following simplified form.

$$P\left(\vec{X}\right)=\mu \left(\vec{X}\right)A\left(\vec{X}\right)$$ (16)

Here, $\mu \left(\vec{X}\right)$ is unknown while $A\left(\vec{X}\right)$ is completely determined by the displacements. At static equilibrium and in the absence of body forces, the conservation of linear momentum requires that the divergence of the first Piola-Kirchhoff stress, $P\left(\vec{X}\right)$ , with respect to the material coordinates, $\vec{X}$ , vanishes.

$$\nabla_{\vec{X}}\cdot P\left(\vec{X}\right)=0$$ (17)

From our earlier simplified form for $P\left(\vec{X}\right)$ , the condition of static equilibrium becomes

$$\nabla_{\vec{X}}\cdot \left(\mu \left(\vec{X}\right)P\left(\vec{X}\right)\right)=0.$$ (18)

In the AWE formulation of the inverse elasticity problem for a linearly elastic material, we seek a variational solution for $\mu \left(\vec{X}\right)$ to the above differential equation. This yields a map $\mu \left(\vec{X}\right)$ of relative stiffnesses, whose scale is determined by the specified-mean boundary condition (Albocher et al., 2009). After solving the inverse problem for $\mu \left(\vec{X}\right)$ , we rescale $\mu \left(\vec{X}\right)$ so that the mean stiffness throughout the domain matches the organ-scale stiffness determined by calibrating the finite-element model.

From Holzapfel’s equation (6.180) (Holzapfel, 2000), which is reproduced below as Eq. 19, along with the strain-energy function mentioned earlier, we determine the components of the elasticity tensor, C , at each state of deformation for each position within the domain. In the following equation, $S_{a}$ are the principal values of the second Piola-Kirchoff stress tensor, $\lambda_{a}$ are the principal stretches, and ${\hat{N}}_{a}$ are the principal directions of the deformation.

$$C=\sum_{a,b=1}^{3}\frac{1}{\lambda_{b}}\frac{\partial S_{a}}{\partial \lambda_{b}}{\hat{N}}_{a}⊗{\hat{N}}_{a}⊗{\hat{N}}_{b}⊗{\hat{N}}_{b}+\sum_{a,b=1,a\neq b}^{3}\frac{S_{b}-S_{a}}{\lambda_{b}^{2}-\lambda_{a}^{2}}\left({\hat{N}}_{a}⊗{\hat{N}}_{b}⊗{\hat{N}}_{a}⊗{\hat{N}}_{b}+{\hat{N}}_{a}⊗{\hat{N}}_{b}⊗{\hat{N}}_{b}⊗{\hat{N}}_{a}\right)$$ (19)

Finally, we perform an iterative optimization to determine the Young’s modulus and Poisson’s ratio fields that optimally approximate the components this tensor. This Young’s modulus is what we ultimately report as the lung’s stiffness (Figure 1D).

### 2.5 Statistical comparison of intergroup and intragroup strains and stiffnesses

To characterize changes in strain and stress fields with pressure, we discretized the domain by coherence length into 50 × 50 pixel patches (See Supplementary Figure S5), the dimensions of which were chosen to minimize the correlation between image patches. We then computed the mean within each patch, along with the standard deviation across patches within the same domain. With these means and standard deviations, *p*-values were computed (i) across image patches within the same field in order to examine the significance of spatial variations in strain and stiffness and (ii) across pressures within the same patch in order to examine the significance of pressure-driven changes in strain and stiffness within a given patch.

## 3 Results and discussion

### 3.1 Validating the finite-element model

We begin by describing and assessing the finite-element model of the organ, which constitutes the first stage of the system. The image classifiers described in the methods yield high-quality segmentations of the lung and the ribcage. These segmentations are subsequently used to construct a realistic, though simplified, model of the lung within the crystal ribcage (Figure 2A). We find that the pressure-stretch curve of the finite-element model changes smoothly with the parameter α of the hyperelastic foam model; from these curves, we construct a smooth surface approximating the transpulmonary pressure as a function of stretch λ and material parameter α (Figure 2B). Having characterized how the finite-element model’s response changes with α , we solve for the values of μ and α that maximize the likelihood of observing the distensions that we measure in the crystal ribcage; for the data collected in the present study, we determine that the optimal value for μ is 0.61 kPa and for α is 12.4 (Figure 2C) through the optimization described earlier. The general form of the hyperelastic foam model consists of multiple additive terms. Although a greater number of terms should in theory lead to a better approximation of the data, fitting a model with multiple terms is complicated by the model’s nonlinearity in the α parameter, so that it is not possible to reuse the calibration surface to fit successive terms beyond the first. For simplicity and because the subsequent outcome is adequate for our purposes, we settle for a single term. These simulations consistently converge to the same numerical equilibrium. We observe that the qualitative distension of the finite-element model matches that of the real lung in the crystal ribcage (Figure 2D). Despite the finite-element model’s homogeneous material properties, its strains (Figure 2E) and stresses (Figure 2F) grow increasingly heterogeneous across the lung’s surface as the pressure increases, with the highest values occurring near the spine.

### 3.2 Validating the multiscale model

**FIGURE 3:**
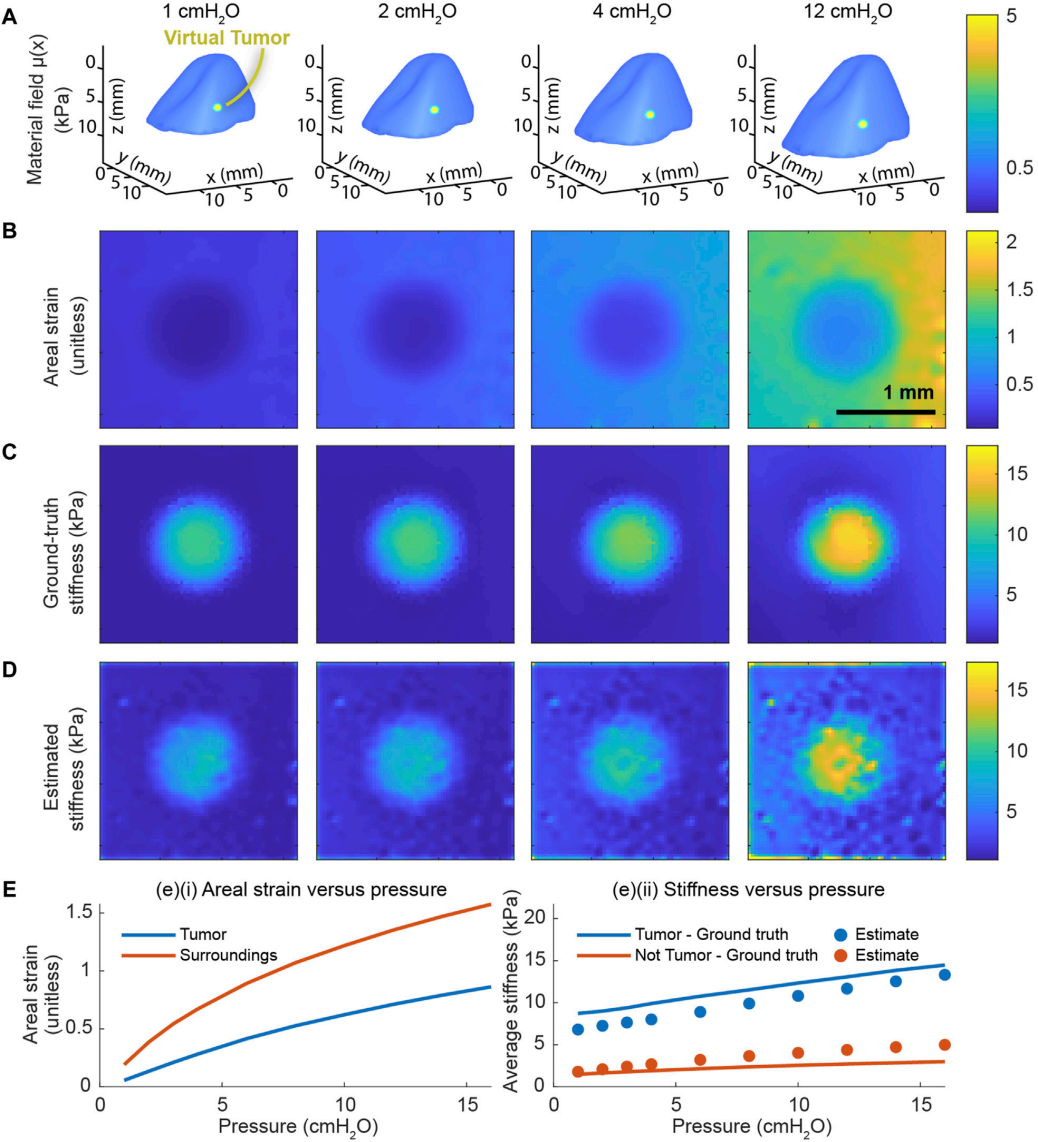
End-to-end validation of the whole-organ and microscale models. The validation includes applying the multiscale model to a finite-element model whose material field contains an inclusion representing a cancerous tumor. **(A)** The material field superposed over the deformed geometry of the finite-element model across a range of distending pressures. **(B)** The total areal strain at a subset of these same pressures. **(C)** The ground-truth stiffness distribution throughout the finite-element model determined from material field, the hyperelastic constitutive equation, and the state of deformation. **(D)** The corresponding stiffnesses in absolute units (kPa) throughout the domain determined by our model based on the simulated displacements. **(E)** The cumulative areal strain and the absolute stiffness change nonlinearly with the distending pressure. **(E)** (i) The average, nominal areal strain inside the tumor is consistently lower than the same outside the tumor. **(E)** (ii) The mean value of the ground-truth stiffness distribution is strongly correlated with the stiffness distribution predicted by the multiscale model.

After constructing the finite-element model and calibrating its material constants, we proceed to evaluate the system’s predictive performance. To do so, we modify the calibrated finite-element model to contain a stiffer inclusion representing a tumor (Figure 3A). From the displacements of this model over a range of pressures, we solve the inverse elasticity problem in the vicinity of the tumor. Across the entire range of distending pressures, we find that the total areal strain (Figure 3B) is highly correlated with the ground-truth stiffness (Figure 3C). We further find that the stiffness estimate produced by our multiscale model (Figure 3D) is well-correlated with the ground truth. Although the prediction is somewhat biased relative to the ground truth, we find that the mean of the predicted stiffness is strongly correlated with the mean of the ground truth both inside and outside the tumor (Figure 3E). In a simpler setting, our preliminary work also indicates that the nonlinear formulation of the inverse elasticity problem accurately predicts the stiffness distribution throughout a 2D, hyperelastic membrane (Supplementary Figure S4). In contrast, our preliminary work also indicates that a piecewise-linear formulation of the inverse elasticity problem fails to do so (Supplementary Figure S5).

### 3.3 Lung stiffness at alveolar resolution

**FIGURE 4:**
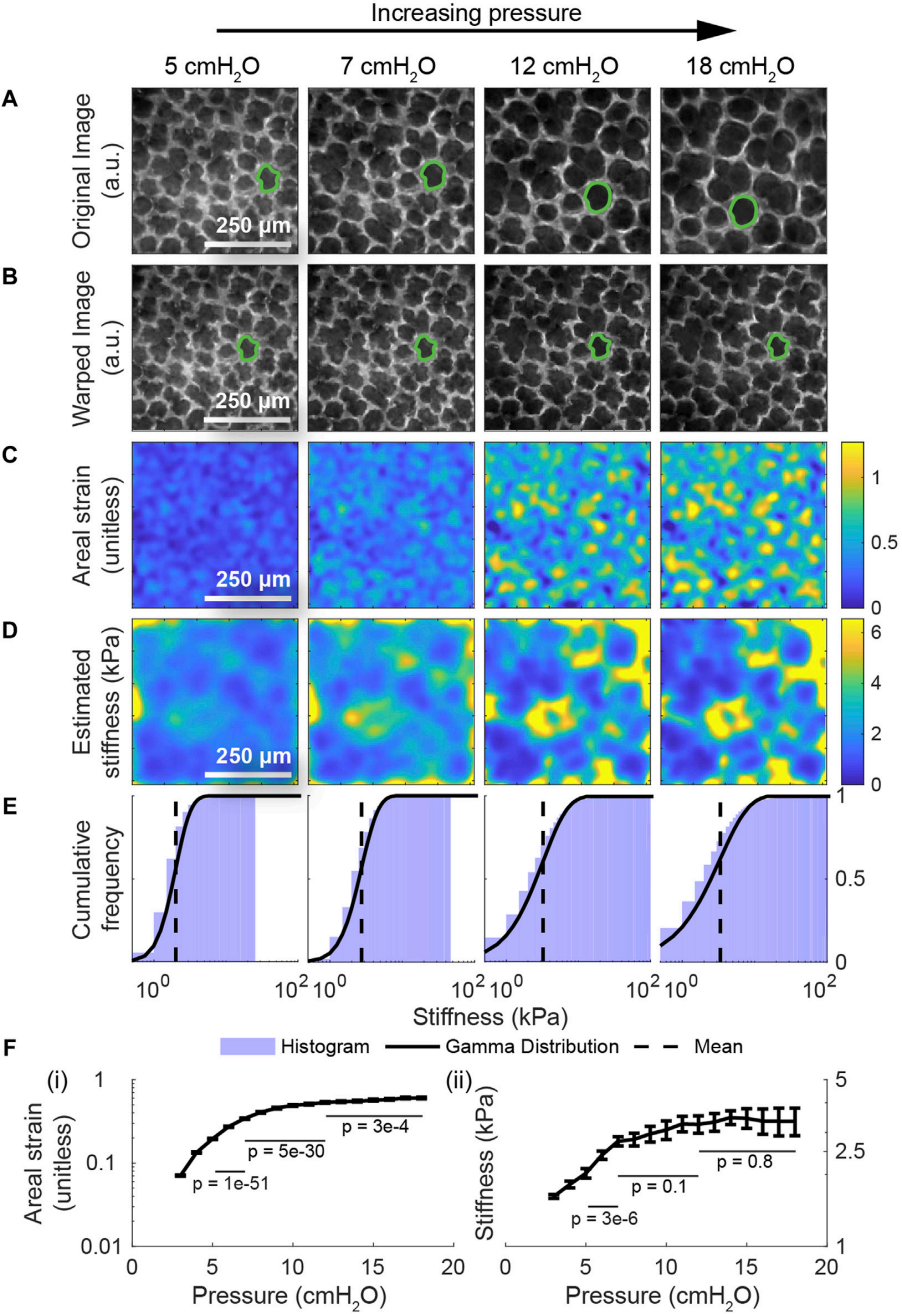
Providing the alveolus-scale stiffness map during the full breathing cycle in the healthy lung. **(A)** The same region of interest within a healthy lung from a transgenic, mTmG mouse expressing the tdTomato fluorescent label at four different distending pressures. **(B)** The result of computationally deforming these images back to the lung’s geometry at 2 cmH_2_O using the registered displacements. The displacement maps used to deform these images were subsequently used as inputs to the multiscale model to determine the distribution of stiffnesses throughout the healthy tissue. **(C)** The areal strain relative to the geometry at 2 cmH_2_O. We observe that the qualitative pattern in the computed strains is largely conserved across all pressures. Row **(D)** depicts the corresponding stiffnesses in absolute units throughout the domain determined by applying our model to these images. As with the areal strain maps, the stiffness maps are qualitatively similar across the whole range of pressures. **(E)** The histogram and corresponding Gamma distribution of the stiffnesses throughout the domain, demonstrating that the mean and the heterogeneity in the lung’s stiffness increase with distension. **(F)** The nonlinear change in mean strain and mean stiffness with the distending pressure. The error bars represent the standard error from the mean, computed by discretizing the domain into a coherence length of 50 × 50 pixel patches (See Supplementary Figure S6). Computed using Student’s t-test, *p*-values are shown for the change in strain (and for the change in stiffness) from 5-7 cmH_2_O, 7–12 cmH_2_O, and 12–18 cmH_2_O; the strains are consistently statistically significant, while the stiffnesses are begin statistically significant and then decrease in significance. We further observe that the variance in the stiffness increases with transpulmonary pressure, which is consistent with our previous finding on relative stiffness (Banerji et al., 2023).

After validating the model, we next apply the model to real images of the lung in the crystal ribcage to measure the stiffness of the lung in absolute units and at alveolar resolution. First, we apply the model to images of the same region of interest in the healthy lung over a range of distending pressures (Figure 4A). To demonstrate the accuracy of the registration, we warp them back to the reference configuration at 2 cmH_2_O (Figure 4B). We observe that the areal strain varies substantially on the length scale of an individual alveolus, with airspaces stretching much more than the septum (Figure 4C). Likewise, we see that the stiffness varies on a similar length scale, and we report for the first time, a noninvasive measurement of the absolute stiffness of the lung’s surface both in the airspace and in the septum; stiffnesses within the airspace are close to 1–2 kPa, while stiffnesses in the septum commonly reach as high as 15 kPa at higher pressures (Figure 4D). These values are consistent with measurements taken using other techniques like atomic-force microscopy (AFM), for which estimates for the lung’s shear modulus commonly range from 0.5 to 3 kPa (Polio et al., 2018; Jorba et al., 2019). Another study (Perlman and Wu, 2014) predicted that the Young’s modulus of the septum ranges from 12 kPa at low transpulmonary pressures to 140 kPa at high transpulmonary pressures; the lower bound is very close to our estimate, while the upper bound is of the same magnitude as ours (Figure 4D). Perhaps most significantly, we observe that the pattern of stiffnesses throughout the domain is conserved across the entire range of pressures. Finally, we note that the mean stiffness of the tissue rises linearly up to 7 cmH_2_O, and then the stiffness quickly plateaus with increasing transpulmonary pressure (Figures 4E, F). Based on the scheme previously described in the methods (Supplementary Figure S6), *p*-values computed using Student’s t-test indicate that the change in strain across pressures is statistically significant with *p* < 0.05 from 5-7 cmH_2_O, 7–12 cmH2O, and 12–18 cmH2O (Figure 4F). On the other hand, the change in stiffness is only statistically significant at lower pressures, owing to higher variance in the stiffness and its apparent plateau at higher pressures (Figures 4E, F). We also predict that the variance in the lung’s stiffness increases with distension (Figure 4F); this prediction is consistent with our previous, independent measurement of the lung’s strain-stiffening behavior from manual measurements of alveolar areas across different pressures (Banerji et al., 2023). To our knowledge, this is the first time that the lung’s stiffness, and its change with distension, has been measured under *ex vivo* conditions with physiologically realistic boundary conditions in absolute units and at alveolar resolution.

**FIGURE 5:**
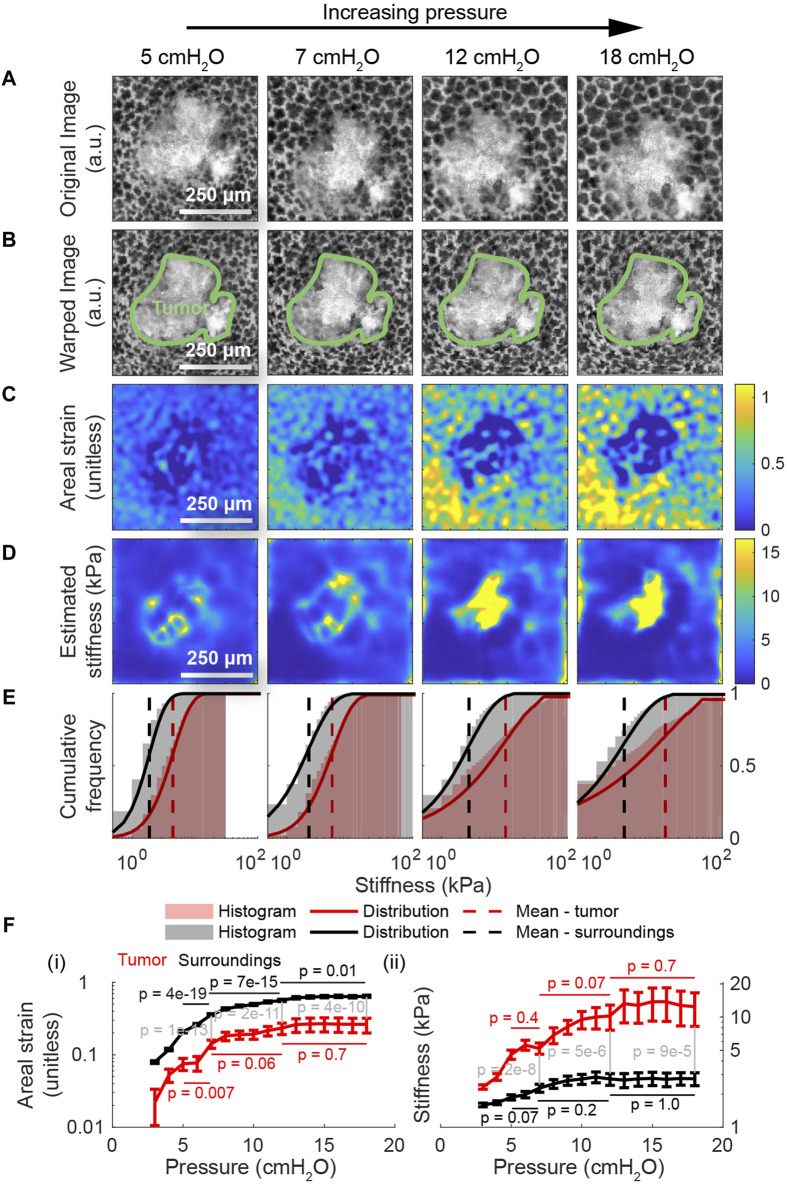
Applying the model to the lung with cancer. **(A)** The same region of interest within a lung presenting with primary cancer from a transgenic, mTmG mouse expressing the tdTomato fluorescent label at four different distending pressures. These images were used as inputs to the multiscale model to determine the distribution of stiffnesses throughout the tumor and its surroundings. **(B)** The result of computationally deforming these images back to the geometry at 2 cmH_2_O using the registered displacements. **(C)** The corresponding areal strain, relative to the geometry at 2 cmH_2_O, induced by the given increase in transpulmonary pressure. As with healthy tissue, the range of areal strains decreases with increasing transpulmonary pressure, indicating an increase in tissue stiffness. Unlike healthy tissue, the tumor clearly exhibits much lower stretch than the surroundings. **(D)** The corresponding stiffnesses in absolute units throughout the domain determined by applying our model to these images. At lower pressures, the tumor is significantly stiffer than its surroundings. The intratumor and extratumor stiffnesses both increase with transpulmonary pressure. **(E)** The stiffness distributions show that the tumor is consistently stiffer surrounding tissue across all pressures, with tumors having higher maximum stiffness and greater variability in stiffness. **(F)** Compared to surrounding tissue, the tumor deforms less with increasing transpulmonary pressure (**(F)** (i)); stiffens more (**(F)** (ii)), notably being 4.8 times stiffer at 18 cmH_2_O; and exhibits greater variance in stiffness, mirroring trends seen in earlier figures. Computed using Student’s t-test, *p*-values are shown for the change in strain (and for the change in stiffness) from 5-7 cmH_2_O, 7–12 cmH_2_O, and 12–18 cmH_2_O. Additionally, *p*-values are shown comparing the strain and stiffness of the lung tissue *versus* the tumor tissue at pressures 7 cmH_2_O, 12 cmH_2_O, and 18 cmH_2_O; the differences between classes are statistically significant for all pressure changes.

Having applied the model to the healthy lung, we do the same for images of the lung with cancer (Figure 5A) to characterize the effect of cancer on the lung’s material properties. As with the previous figure, the second row (Figure 5B) shows the result of deforming these images back to the geometry of the lung at 2 cmH_2_O using the registered displacements. Here, we observe that the strain inside the tumor is substantially lower than the strain outside the tumor across all measured pressures (Figure 5C). Consistent with these observations, the estimated stiffness inside the tumor is substantially higher than the stiffness outside at lower pressures (Figure 5D). Whereas the majority of stiffnesses in the healthy lung are below 5 kPa at pressures up to 10 cmH_2_O, a large fraction of the tumor exceeds these stiffnesses at these same pressures (Figure 5F). Using the method described previously (Supplementary Figure S6), *p*-values computed using Student’s t-test indicate that the change in strain across pressures within the lung tissue is statistically significant with *p* < 0.05 from 5-7 cmH_2_O, 7–12 cmH2O, and 12–18 cmH2O, while changes in the strain within the tumor are insignificant at 7–12 cmH2O and 12–18 cmH2O (Figure 5F). Once again, in both types of tissue, the change in stiffness becomes insignificant at higher pressures. On the other hand, at the same pressure, the difference in strain and stiffness across groups (i.e., lung or tumor) is statistically significant with *p* < 0.05 across all pressures.

Relative to the healthy case, we also observe that the stiffness of the tissue outside the tumor is depressed by about 20%, suggesting that the tumor may remodel the lung even in regions that are not visible by light microscopy alone; whether that remodeling is due to the tumor visible in the images or due to other tumors that are below the lung’s surface is not clear. Moreover, while the mean stiffness of the lung tissue increases in nonlinear fashion as in the case of the healthy lung, the mean stiffness of the tumor increases much more quickly (Figure 5E). In summary, this measurement represents the first measurement of the absolute stiffness of a lung tumor under physiologically realistic boundary conditions and at alveolar resolution, and we see evidence of remodeling beyond the visible bounds of the tumor. (Supplementary Figures S7–S9) depict these same maps across the entire range of transpulmonary pressures.

### 3.4 A hypothesis explaining the experimental data

To explain the preceding observations, we briefly reflect on the biochemical structures and physical principles that determine the material properties of the lung and solid tumors. The primary load-bearing elements of the extracellular matrix are elastin, collagen type-1, and collage type-3 (https://www.frontiersin.org/journals/network-physiology/articles/10.3389/fnetp.2023.1142245/full) (Shi et al., 2022). Although two isolated collagen helices with the same geometric configuration should exhibit identical material properties, determined by the interplay between intramolecular and intermolecular forces between monomeric subunits of the triple helix (In’T Veld and Stevens, 2008), it is well-known that collagen arranges itself into more complex, hierarchical structures (Fratzl, 2008). Within these structures, greater cross-linking between individual collagen helices increases the stiffness at the tissue scale (Fratzl, 2008). Furthermore, given that entropic effects largely dominate in determining the material properties of rubber-like polymer networks, the current geometric configuration of a polymer network influences its current stiffness (James and Guth, 1944; Treloar, 1974; Holzapfel and Simo, 1996; Boyce and Arruda, 2000; Treloar, 2005). Finally, recalling that networks of parallel springs are stiffer than networks of springs in series (In’T Veld and Stevens, 2008), we observe that the stiffness of such a network largely depends on its topology. Variations in any of these three contributors can therefore lead to variations in tissue stiffness at cellular, alveolar, and organ length scales.

Based on the biochemical structure of the extracellular matrix, there are thus four obvious reasons why the tumor should be stiffer than the surrounding tissue. First, unlike the healthy lung which contains airspaces, solid tumors are generally aggregates of cells and extracellular matrix lacking holes or gaps at the cellular length scale; their simply connected structure therefore elevates their stiffness relative to the multiply connected structure of the healthy parenchyma. Second, even if we ignore the airspaces, pathologically elevated deposition of extracellular matrix within the tumor increases the matrix’s density compared to healthy tissue, and greater density is naturally associated with elevated stiffness essentially because there are more load-bearing elements at the molecular level (Nia et al., 2020a). Third, pathologically elevated cross-linking also increases the stiffness of the extracellular matrix. Fourth, while the polyhedral topology of the parenchyma essentially forces the alignment of collagen fibrils within the mid-plane of the septum and reduces the entropy of the extracellular matrix, the collagen fibrils within solid tumors can be arranged in arbitrary orientations; stretching a solid tumor by the same distance should therefore affect the quantity of work required to produce the same stretch.

Next, we discuss the physical source of strain stiffening. First, we note that statistical thermodynamics predicts that single polymer molecules exhibit strain-stiffening behavior; as the molecule stretches, the number of available geometric configurations decreases, the change in entropy between successive states of elongation increases, and thus the force required to produce the same distension monotonically increases with stretch (James and Guth, 1944; Treloar, 1974; Beatty, 1987; Holzapfel and Simo, 1996). Indeed, recent Steered Molecular Dynamics simulations of individual collagen helices predicted that these polymers exhibit strain-stiffening, with their stiffnesses ranging from 5 kPa to 15 kPa (In’T Veld and Stevens, 2008). At higher levels of organization, AFM studies confirm that individual collagen fibrils also grow stiffer with strain (Sherman et al., 2015; Halvorsen et al., 2023), and that collagen-based biomaterials likewise exhibit the same behavior (Fratzl, 2008; Sherman et al., 2015). Most likely, then, the strain-stiffening behavior at the tissue scale directly follows from the strain-stiffening behavior of individual collagen helices at the molecular scale. This is, essentially, the central hypothesis underpinning classical derivations of constitutive equations for hyperelastic materials, and it motivates our choice of a hyperelastic material law (Vawter et al., 1979; Boyce and Arruda, 2000).

The theory of percolation (Suki and Bates, 2011), which predicts that stretch produces gradual straightening and alignment of initially wave collagen fibers, may seem to imply that the lung’s stiffness should grow increasingly homogeneous with increasing stretch. But our observation that stiffness heterogeneity increases with pressure, an effect referred to as heteroscedasticity in the statistics literature (Bishop, 2006), directly contradicts this hypothesis. Several studies agree with our prediction across multiple length scales. First, studies on the strain-stiffening behavior of individual collagen fibrils have also reported that the variance in fibril stiffness increases with strain (Sherman et al., 2015; Halvorsen et al., 2023). Second, at the alveolar length scale, both spring-network studies (Maksym et al., 1998; Cavalcante et al., 2005) and finite-element analysis studies (Sarabia-Vallejos et al., 2019) have consistently predicted an increase in septal stiffness heterogeneity with pressure. Finally, at the organ scale, registration-based studies (Mariappan et al., 2014; Mariano et al., 2020) have reported the same. However, one recent multiscale, AFM study (Jorba et al., 2019) measured heteroscedasticity in macroscopic slices of decellularized lung tissue, but that same study did not observe the same trend for microscopic slices. One possible explanation for this disagreement is that tissue resection disrupts this phenomenon. Another possible explanation is that our method computes the variance over the stiffness both in the airspace and in the septum, but that does not explain why the aforementioned studies have reported the same phenomenon. Although we cannot discount that the geometric configuration of the individual polymers within the network somehow contributes to this behavior, the heteroscedasticity at the tissue scale may arise, at least in part, from the heteroscedasticity of the individual collagen fibrils comprising the extracellular matrix. The precise explanation for the source of heteroscedasticity at the fibril scale is beyond the scope of this work.

### 3.5 Addressing assumptions in our model

Although these findings represent a significant advance in our ability to quantify the lung’s mechanical properties at cellular resolution in both health and disease, the model makes several simplifying assumptions that we should acknowledge. These assumptions, which vary in the magnitude of their potential impacts, include (i) approximating the Jacobian as ${\left(\lambda_{1}\lambda_{2}\right)}^{1.5}$ when solving the inverse elasticity problem, (ii) adopting the same constitutive equation at the alveolar and organ length scales, (iii) assuming that the Poisson ratio is 0.2 at both length scales, (iv) neglecting higher-order features of the lung, such as interlobular fissures and airways, when constructing the finite-element model’s geometry, (v) assuming that the lung’s stiffness is generally homogeneous at the organ scale, (vi) treating the airspace as a tensile element when solving the inverse problem, (vii) assuming isotropicity when solving for the Young’s modulus, and (viii) neglecting the influence of gravity on the lung’s deformation.

In Supplementary Figure S8, assumption (i) was shown to exert a relatively minor effect, typically introducing less than 10% error into our approximation of the Jacobian determinant. To address assumption (ii), we observe that the extracellular matrix is the primary determinant of the lung’s material properties across all length scales (https://www.frontiersin.org/journals/network-physiology/articles/10.3389/fnetp.2023.1272172/full, https://www.frontiersin.org/journals/network-physiology/articles/10.3389/fnetp.2024.1396383/full). While studies have shown that the lung’s macroscale stiffness is significantly less than the stiffness of individual lung cells (Sicard et al., 2018), owing to the porosity of the tissue, our approach ensures that the average stiffness, computed across many alveoli over both the airspace and the septum, matches the organ-scale stiffness. As the number of alveoli in the calculation approach the total number of alveoli in the lung, this average must approach the whole lung’s average stiffness. Consequently, we argue it is reasonable to enforce equality between these quantities. This same reasoning also justifies assumption (iii).

Assumption (iv) is, in part, justified on the basis that parenchyma comprises over 95% of the lung’s total volume (West and Luks, 2020), which implies that the effect of stiffer airways on the deformation should be small in the distal parts of the lung. On the other hand, evidence suggests (Lee et al., 1983) that the interlobular fissures relieve stress that may develop on the surface of the lung in their absence. This assumption, therefore, may affect the accuracy depending on the proximity to a fissure.

Assumption (v) is challenged by MRE studies (Marinelli et al., 2017) that reveal significant regional variation in lung compliance at the organ scale. For healthy individuals, this study reported that the mean shear modulus was 0.849 ± 0.250 kPa at residual volume and 1.33 ± 0.195 kPa at total lung capacity. Consequently, the standard deviation decreases from about 30% of the mean value at residual volume to about 15% of the mean at total lung capacity. Thus, we may expect our stiffness maps to incur similar errors due to this assumption. To model the effect of regional variations in lung stiffness, future studies may adapt this model in two ways, either (a) modeling spatial heterogeneity in parenchymal stiffness by adjusting the material field modeling (e.g., Figures 3A, B) finer geometric features like fissures and conducting airways. Because our images are collected within microns of the pleural surface, we suspect that the airspace region in the images effectively exhibits some resistance to deformation, justifying assumption (vi); even if the airspace lacks effective stiffness, that should be reflected by a low value in the stiffness mapping, which is exactly what is shown in Figures 4, 5. Finally, in support of assumption (vii), we performed simulations of the finite-element model with an embedded inclusion, and we found that assuming isotropicity (Supplementary Figure S10) led to similar results as assuming orthotropicity (Supplementary Figure S11), with both models in reasonable agreement with the ground truth (Supplementary Figure S12).

Assumption (viii), the decision to neglect gravity in modeling the lung, was addressed previously in the methods. Briefly, although previous studies (Tawhai et al., 2009; Shi et al., 2022) have shown that gravity significantly influences the mechanics of the human lung, similar studies have not been done in the mouse. We offer two arguments, however, in support of our position. First, these previous studies on the human lung suggest that gravity is insignificant to the solid mechanics of the mouse lung. One recent theoretical analysis (Shi et al., 2022) on the human lung distilled the influence of gravity on alveolar mechanics to the weight of tissue below a given alveolus; consequently, the study found that the effects of gravity are more significant near the apex than the base. Because the weight of the mouse lung is about 1 G, and because the stiffness of the mouse lung is similar to that of the human lung, its weight according to this model should not significantly influence its mechanical behavior. Using CT images to measure regional variations in lung density, another study (Tawhai et al., 2009) showed that gravity causes the density of the human lung to increase linearly with vertical displacements toward the Earth; critically, the study showed that lung density only changes by a few percent of the mean with displacements near 1 cm. Because the density and stiffness of the mouse lung is similar to that of the human lung, we likewise expect gravity to have only a modest influence on tissue density in the mouse. Second, as described earlier in the methods, dimensional analysis (Munson et al., 2012) reveals that the stresses developed within the mouse lung are significantly larger than the hydrostatic stresses arising due to gravity.

## 4 Conclusion

We have built the first model capable of measuring the absolute stiffness of the lung at microscale resolution and under physiologically realistic boundary conditions. We have shown that our model can measure the nonlinear stiffening of the lung with increasing stretch, and that the relative stiffness distribution throughout the domain is, at least in the case of the healthy lung, largely conserved across a range of pressures, giving further confidence that our prediction corresponds to reality since these stiffness maps have been produced by completely different displacement maps. Furthermore, we have shown that our model’s quantitative predictions are consistent with state-of-the-art measurements based on AFM. Finally, we have demonstrated the capability of our model to identify and measure the stiffness of tumors within the lung tissue. Here, we have shown, for the first time, that the tumor exhibits similar strain-stiffening behavior to the lung tissue itself, but that the tumor stiffens more substantially than the surroundings; in the state of greatest distension, for example, the tumor’s mean stiffness is 4.8 times greater than that of the surroundings. Additionally, because the variance in the stiffness increases with transpulmonary pressure, we have shown that the heterogeneity in the stiffness distribution likewise increases with pressure, with greater heterogeneity in the tumor than in the surroundings. Although applied here to a mouse model of the lung, the theoretical framework introduced in this study is applicable to other species and to other imaging modalities. In the research setting, for example, the crystal ribcage and this analytic methodology may be applied to transplant-rejected human lungs, which would in turn enable the first real-time visualization and mechanical characterization of the human lung at alveolar resolution across its entire surface. In the clinical setting, the model could be calibrated against CT or MRI images of the lung during the ventilation cycle, allowing clinicians to accurately and quantitatively measure the stiffness of solid tumors for diagnosis, staging, evaluation of treatment response, and longitudinal monitoring of disease progression.

## Data Availability

The raw data supporting the conclusion of this article will be made available by the authors, without undue reservation.
