# Supplementary material for: Mapping the strain-stiffening behavior of the lung and lung cancer at microscale resolution using the crystal ribcage
Source: Front Netw Physiol. 2024 Jul 10;4:1396593. doi: 10.3389/fnetp.2024.1396593 (PMC11266057; doi:10.3389/fnetp.2024.1396593)
Supplement: Supplementary file 1 [file Table1.docx]

**Supplementary Materials**


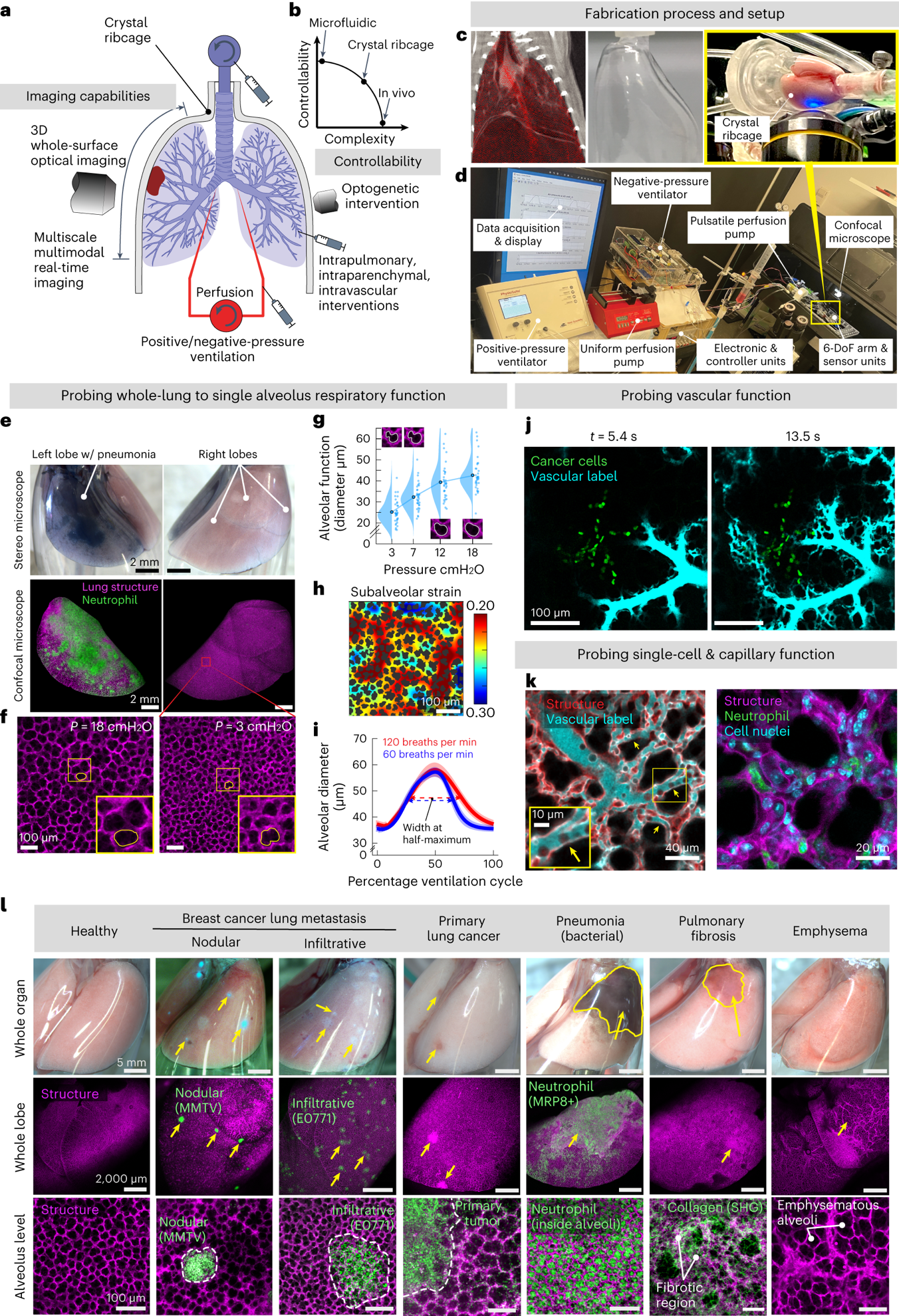


**Figure S1 | Illustration of the crystal ribcage platform.** This figure reproduces panels (a-d) from Figure 1 in our earlier work^1^ and depicts the details of the crystal ribcage platform. The panels are as follows: (a) Schematic of the lung within the crystal ribcage, depicting the imaging, controllability, and intervention capabilities of the platform, (b) The crystal ribcage supports the same imaging capabilities and controllability of organ-on-chip models, while maintaining the complex environment of *in vivo* lungs, (c) Age- and strain-specific microCT scans are used to fabricate the crystal ribcage through a multistep fabrication process, in which the geometry is derived from microCT images, and (d) The portable platform to maintain, monitor and record the lung physiological condition during real-time imaging.


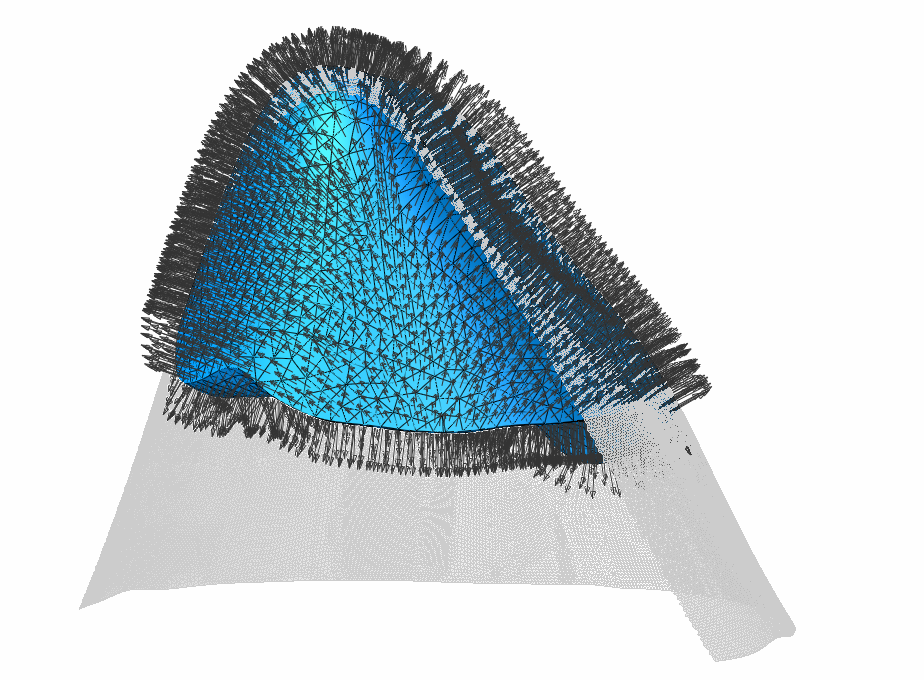


**Figure S2 | The boundary conditions on the finite-element model.** In the simulation, the lung (blue part) is constrained to slide frictionlessly along the surface of the rigid ribcage (gray surface). A negative pressure load (black vectors) is applied to the surface of the lung in order to emulate negative transpulmonary pressure. Part of the virtual ribcage has been visually hidden to show the lung below.


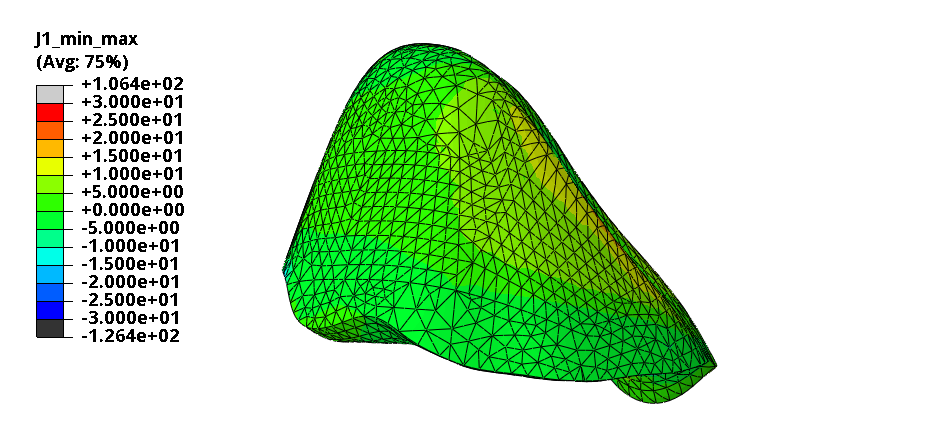

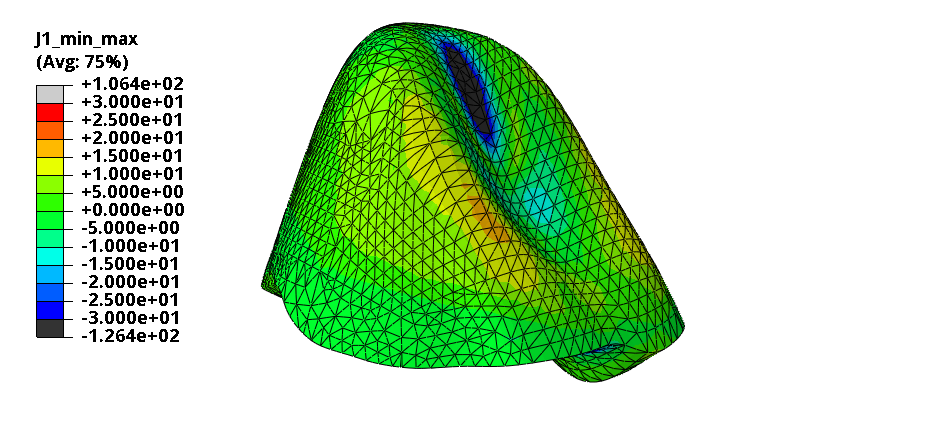

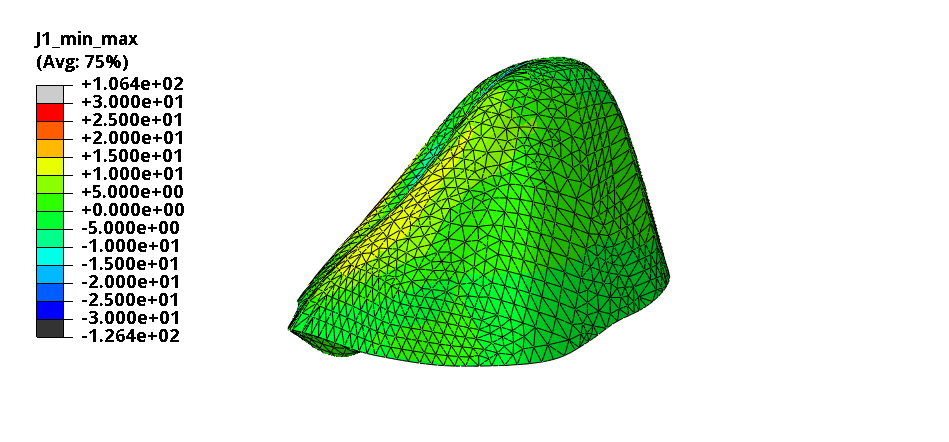


**Figure S3 | Demonstrating the percent error in our pseudo-3D approximation of the Jacobian determinant when solving the inverse problem.** These panels show the percent error of ${{(\lambda}_{1}\lambda_{2})}^{1.5}$ relative to the true Jacobian $\lambda_{1}\lambda_{2}\lambda_{3}$. We see that the approximation is generally accurate to within 5-15% error across the majority of the lung’s surface.

**
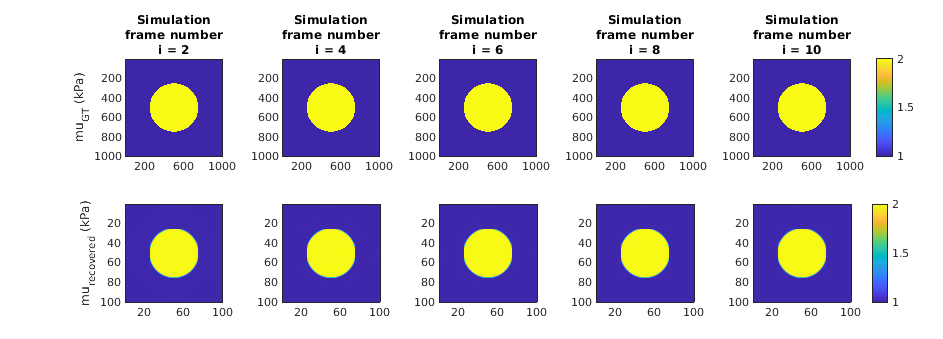
**

**Figure S4 | Validating the nonlinear inverse solver on a simple, pseudo-3D, hyperelastic foam membrane.** In Abaqus, we construct a 2D, hyperelastic foam membrane having a shear modulus of 2 kPa within a circular inclusion and having a shear modulus of 1 kPa outside the inclusion. We then stretch the model by 10% along the two directions parallel to the edges of the domain. The first row shows the ground-truth shear modulus distribution, while the second row provides the shear-modulus distribution estimated from the simulated total displacements and the nonlinear formulation of the inverse solver.

**
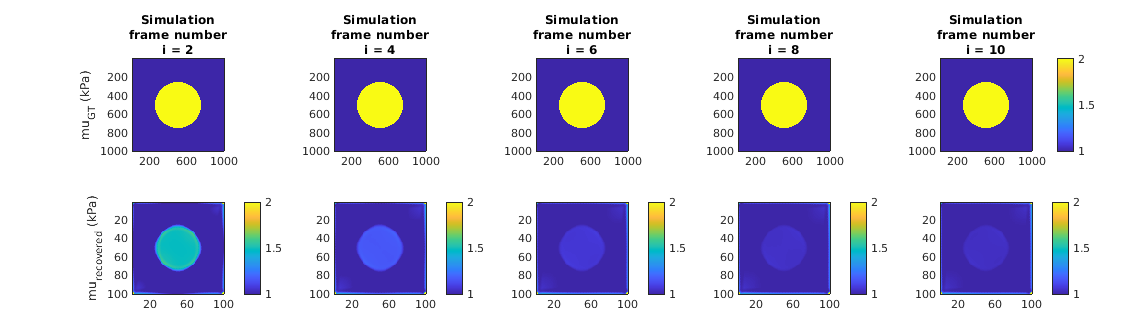
**

**Figure S5 | Assessing the piecewise-linear inverse solver on a simple, pseudo-3D, hyperelastic foam membrane.** In Abaqus, we construct a 2D, hyperelastic foam membrane having a shear modulus of 2 kPa within a circular inclusion and having a shear modulus of 1 kPa outside the inclusion. We then stretch the model by 10% along the two directions parallel to the edges of the domain. The first row shows the ground-truth shear modulus distribution, while the second row provides the shear-modulus distribution estimated from the simulated incremental displacements and the piecewise-linear formulation of the inverse solver.

**
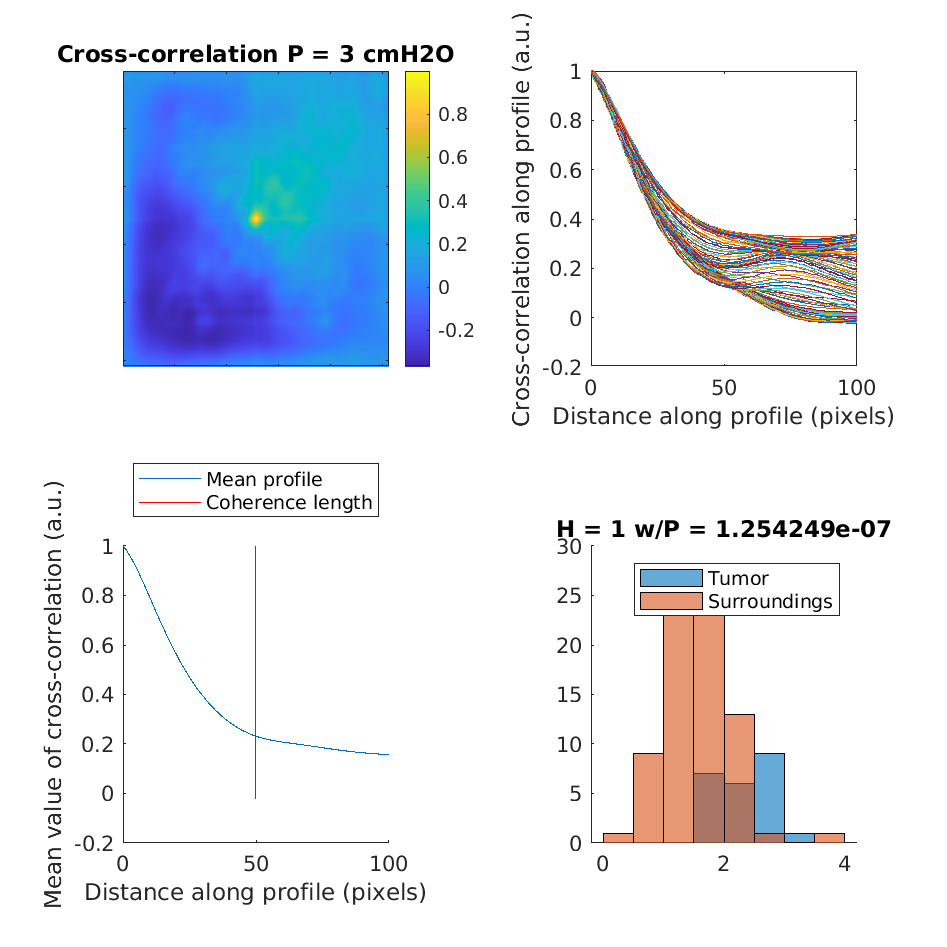
**

**Figure S6 | Determining the coherence length for statistically comparing mappings.** (Top left) Normalized autocorrelation of the estimated stiffnesses for the lung with cancer (Fig. 5). (Top right) 100 profiles of the autocorrelation decay with the magnitude of the displacement of the copy of the original mapping. (Bottom left) The mean profile begins to plateau when the distance between the field and its displaced copy reaches 50 pixels, indicating that 50x50 patches within the image are mutually independent. (Bottom right) The histogram of the stiffnesses within the tumor and the surroundings after discretization of the stiffness mapping into 50x50 pixel patches. The difference between these distributions is statistically significant with a p-value of 1.25e-7.


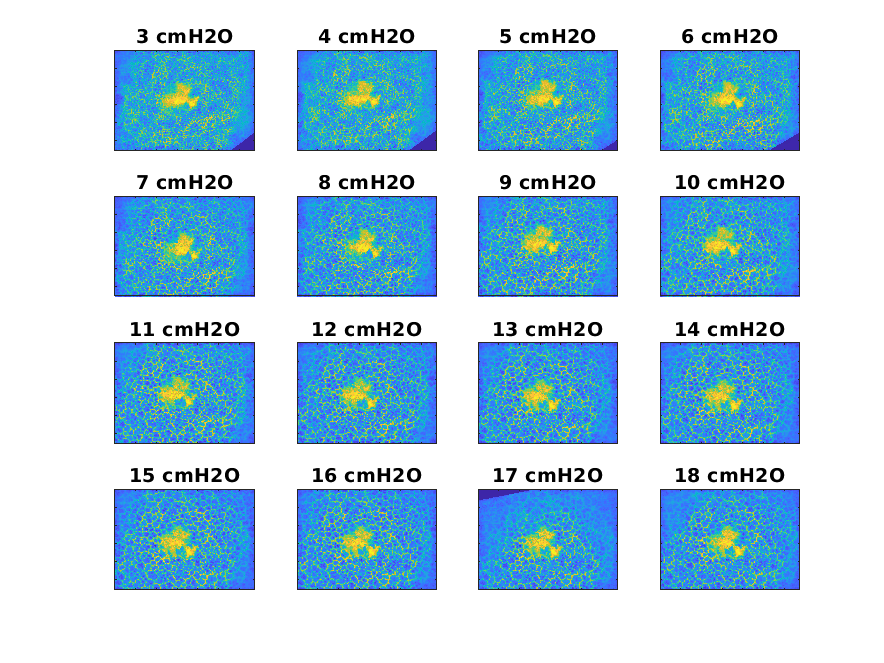


**Figure S7 | Images of the lung from 2 cmH_2_O to 18 cmH_2_O.** During the course of our experiments, we collect images of the lung at pressures incrementing from 2 cmH_2_O to 18 cmH_2_O in increments of 1 cmH_2_O.

**
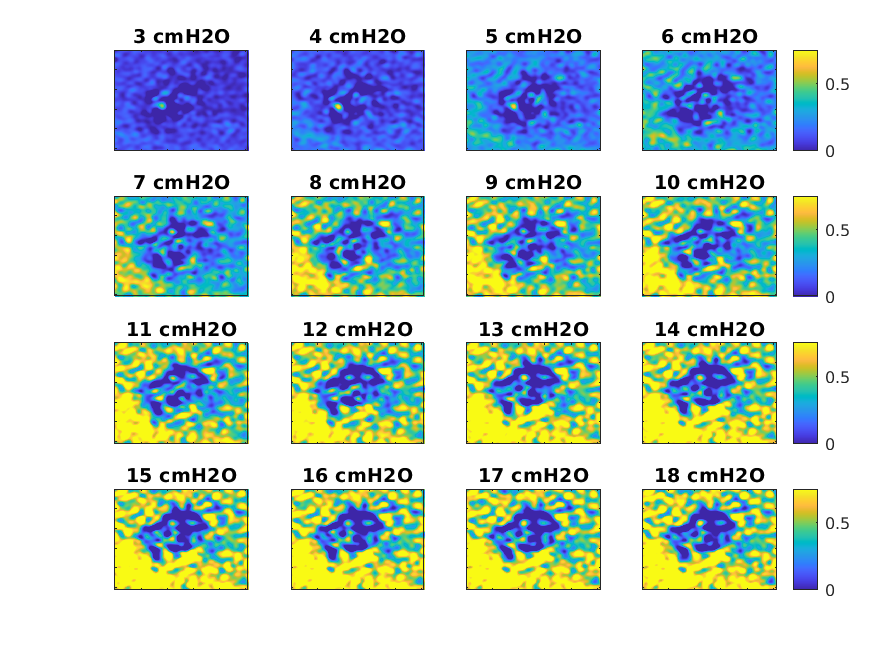
**

**Figure S8 | Strain maps from 2 cmH_2_O to 18 cmH_2_O.** We register images of the lung between consecutive pressures and then compose the resulting displacement mappings. From the composed displacement mappings, we compute the total areal strain throughout the domain at each pressure.

**
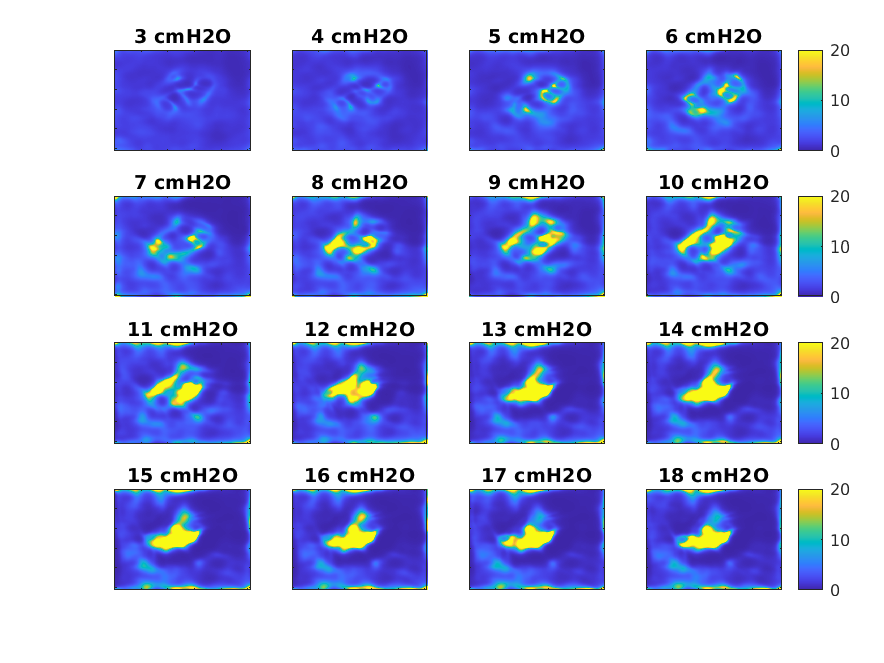
**

**Figure S9 | Stiffness maps from 2 cmH_2_O to 18 cmH_2_O.** From the registered displacements, we also solve the inverse elasticity problem for the stiffnesses throughout the domain at each pressure.


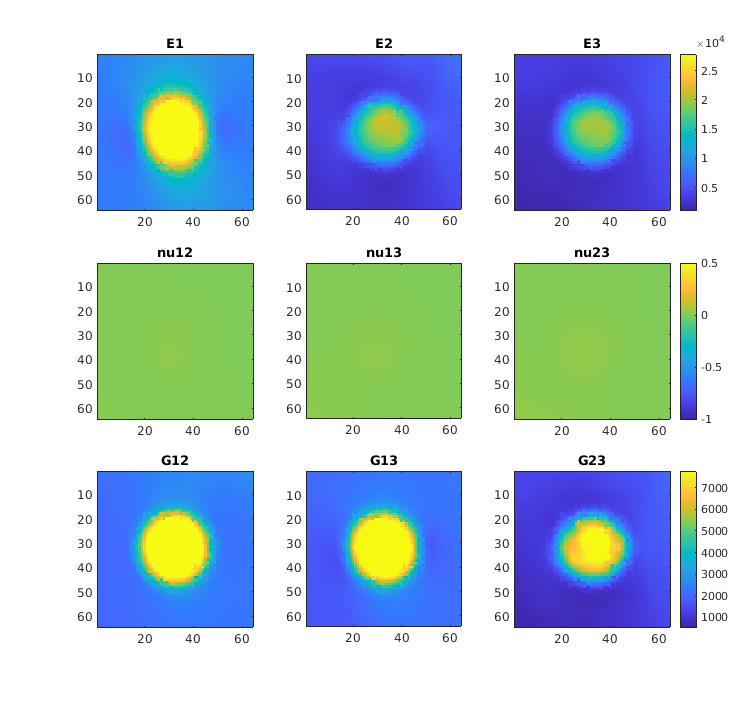


**Figure S10 | The ground-truth, orthotropic stiffness of the finite-element model at 18 cmH_2_O.** As shown in Fig. 3, we simulated distension of the finite-element model containing a stiff inclusion representing a cancerous tumor. Here, we show the ground-truth coefficients characterizing the elasticity tensor at 18 cmH_2_O for comparison to the stiffness maps in S10 and S11. Units of the elasticity moduli and the shear moduli are in Pascals, while the Poisson ratios are unitless.


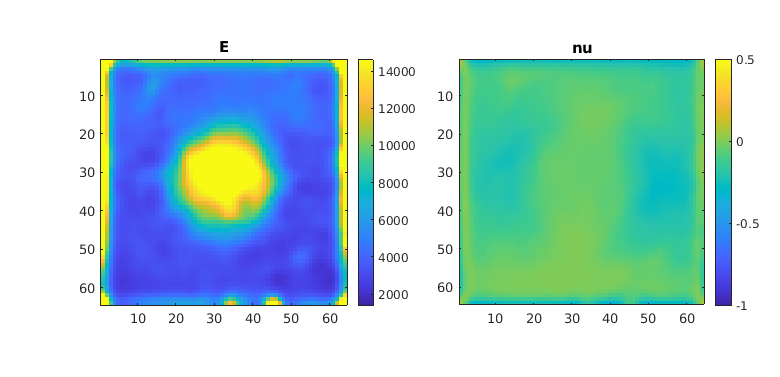


**Figure S11 | The isotropic stiffness of the finite-element model at 18 cmH_2_O.** As shown in Fig. 3, we simulated distension of the finite-element model containing a stiff inclusion representing a cancerous tumor. Here, we show the estimates isotropic coefficients approximating the elasticity tensor at 18 cmH_2_O. Units of the elastic modulus are Pascals, while the Poisson ratio is unitless.


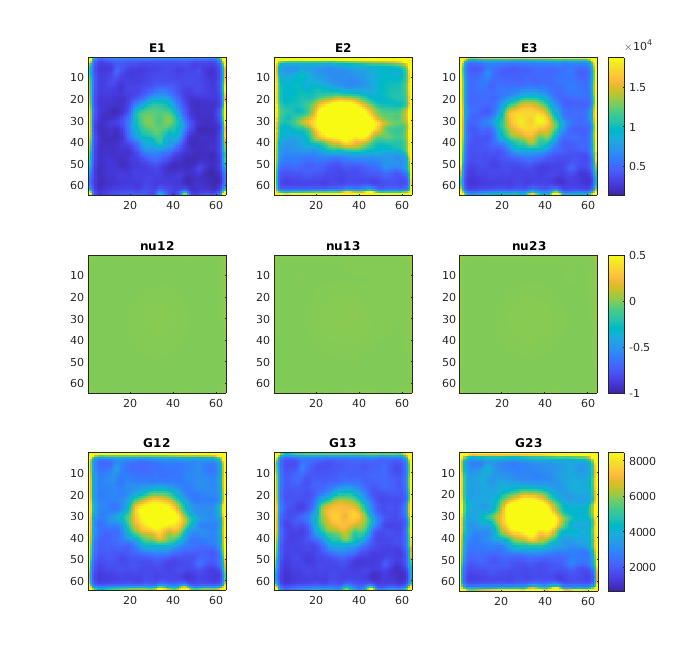


**Figure S12 | The estimated, orthotropic stiffness of the finite-element model at 18 cmH_2_O.** As shown in Fig. 3, we simulated distension of the finite-element model containing a stiff inclusion representing a cancerous tumor. Here, we show the estimated orthotropic coefficients of the elasticity tensor at 18 cmH_2_O. Units of the elastic moduli and the shear moduli are in Pascals, while the Poisson ratios are unitless.
